# Loss of Ranbp2 in motoneurons causes disruption of nucleocytoplasmic and chemokine signaling, proteostasis of hnRNPH3 and Mmp28, and development of amyotrophic lateral sclerosis-like syndromes

**DOI:** 10.1242/dmm.027730

**Published:** 2017-05-01

**Authors:** Kyoung-in Cho, Dosuk Yoon, Sunny Qiu, Zachary Danziger, Warren M. Grill, William C. Wetsel, Paulo A. Ferreira

**Affiliations:** 1 Department of Ophthalmology, Duke University Medical Center, Durham, NC 27710, USA; 2 Department of Biomedical Engineering, Duke University, Durham, NC 27710, USA; 3 Departments of Psychiatry and Behavioral Sciences, Cell Biology, and Neurobiology, Mouse Behavioral and Neuroendocrine Analysis Core Facility, Duke University Medical Center, Durham, NC 27710, USA; 4 Department of Pathology, Duke University Medical Center, Durham, NC 27710, USA

**Keywords:** Ran-binding protein 2, Chemokine signaling, Transcriptomics, Proteostasis, Motoneuron, Mouse gene knock-out, Nucleocytoplasmic transport, Metalloproteinase, Amyotrophic lateral sclerosis

## Abstract

The pathogenic drivers of sporadic and familial motor neuron disease (MND), such amyotrophic lateral sclerosis (ALS), are unknown. MND impairs the Ran GTPase cycle, which controls nucleocytoplasmic transport, ribostasis and proteostasis; however, cause-effect mechanisms of Ran GTPase modulators in motoneuron pathobiology have remained elusive. The cytosolic and peripheral nucleoporin Ranbp2 is a crucial regulator of the Ran GTPase cycle and of the proteostasis of neurological disease-prone substrates, but the roles of Ranbp2 in motoneuron biology and disease remain unknown. This study shows that conditional ablation of *Ranbp2* in mouse Thy1 motoneurons causes ALS syndromes with hypoactivity followed by hindlimb paralysis, respiratory distress and, ultimately, death. These phenotypes are accompanied by: a decline in the nerve conduction velocity, free fatty acids and phophatidylcholine of the sciatic nerve; a reduction in the g-ratios of sciatic and phrenic nerves; and hypertrophy of motoneurons. Furthermore, Ranbp2 loss disrupts the nucleocytoplasmic partitioning of the import and export nuclear receptors importin β and exportin 1, respectively, Ran GTPase and histone deacetylase 4. Whole-transcriptome, proteomic and cellular analyses uncovered that the chemokine receptor Cxcr4, its antagonizing ligands Cxcl12 and Cxcl14, and effector, latent and activated Stat3 all undergo early autocrine and proteostatic deregulation, and intracellular sequestration and aggregation as a result of Ranbp2 loss in motoneurons. These effects were accompanied by paracrine and autocrine neuroglial deregulation of hnRNPH3 proteostasis in sciatic nerve and motoneurons, respectively, and post-transcriptional downregulation of metalloproteinase 28 in the sciatic nerve. Mechanistically, our results demonstrate that Ranbp2 controls nucleocytoplasmic, chemokine and metalloproteinase 28 signaling, and proteostasis of substrates that are crucial to motoneuronal homeostasis and whose impairments by loss of Ranbp2 drive ALS-like syndromes.

## Abstract (precis)

**Summary:** Loss of Ranbp2 in spinal motoneurons drives ALS syndromes in mice and Ranbp2 functions in nucleocytoplasmic trafficking, proteostasis and chemokine signaling uncover novel therapeutic targets and mechanisms for motoneuron disease.

## INTRODUCTION

Motoneuron disease (MND) encompasses neurodegenerative disorders of familial and sporadic origins that affect predominantly motoneurons, but they have varied syndromic presentations (Finsterer and Burgunder, 2014). Although MND is largely sporadic, familial forms of MND, such as familial ALS, are genetically heterogeneous (Cirulli et al., 2015; Robberecht and Philips, 2013). Genetic dissection of familial ALS showed that the molecular players causing ALS typically present pleiotropic functions and broad tissue expression, but motoneurons of the spinal cord and motor cortex are prominently susceptible to neural dysfunction and degeneration (Kiernan et al., 2011). The molecular bases of the selective vulnerability of motoneurons to genetically heterogeneous ALS mutations, and environmental stressors that possibly contribute to sporadic ALS, are poorly understood. Regardless, mounting evidence in mice and humans indicates that sporadic and familial ALS promote impairments of multiple components that are dependent on the Ran GTPase cycle (Jovičić et al., 2015; Freibaum et al., 2015; Zhang et al., 2015; Kim et al., 2013; Boeynaems et al., 2016; Xiao et al., 2015a; Zhang et al., 2006; Kinoshita et al., 2009), which controls nucleocytoplasmic trafficking of substrates implicated in ribostasis (Kim et al., 2013; Dickmanns et al., 2015; Cautain et al., 2015). These impairments lead to pathological imbalances in ribostasis, protein homeostasis (also known as ‘proteostasis’) and toxic aggregation of selective shuttling substrates that are thought to lead ultimately to motoneuron dysfunction and degeneration (Ramaswami et al., 2013; Ling et al., 2013). However, some mouse models of ALS that affect components of the Ran GTPase cycle do not cause motoneuron degeneration for reasons that remain to be elucidated (Koppers et al., 2015; Peters et al., 2015; O'Rourke et al., 2015, 2016).

The Ran-binding proteins 1 (Ranbp1) and 2 (Ranbp2) are the only two known high-affinity binding targets of Ran GTPase (Villa Braslavsky et al., 2000; Vetter et al., 1999; Bischoff et al., 1995; Geyer et al., 1999). Among other unrelated structural and functional domains, Ranbp2 has several interspersed Ran GTPase-binding domains (RBDs) (Wu et al., 1995; Yokoyama et al., 1995; Ferreira et al., 1995). The RBDs of Ranbp2 together with RanGAP promote the hydrolysis of Ran-GTP (Villa Braslavsky et al., 2000; Vetter et al., 1999). Although Ranbp1 is conserved between yeast and humans, Ranbp1 is not part of the nuclear pore complex (NPC) and is not essential in vertebrates (Nagai et al., 2011; Strambio-De-Castillia et al., 2010). By contrast, Ranbp2 is not evolutionarily conserved (Ciccarelli et al., 2005) but is vital to vertebrates (Dawlaty et al., 2008; Aslanukov et al., 2006). Ranbp2 is a large, cytosolic and peripheral nucleoporin (also called Nup358) that comprises cytosolic filaments attached to the NPC (Delphin et al., 1997). Ranbp2 is thought to control the terminal and initial steps of nuclear export and import, respectively, by hydrolyzing and disassembling Ran-GTP bound to binary complexes comprising the nuclear export receptor, Crm1/exportin-1 and nuclear cargoes, and by releasing the nuclear import receptor, importin β, from Ran-GTP upon nuclear export. However, mounting physiological and genetic studies support the notion that Ran GTPase-dependent processes regulated by Ranbp2 harbor unique cell type-restricted functions. The cell-type selective functions of Ranbp2 likely stem from the control of nucleocytoplasmic shuttling, proteostasis or post-translational modifications of cell-type selective, stress-elicited or disease-prone substrates, such as hnRNPA2B1, parkin, M-opsin and Stat3, by unrelated domains of Ranbp2 that are interspersed between its RBDs (Cho et al., 2015a, 2014; Patil et al., 2014; Um et al., 2006; Wälde et al., 2012; Hamada et al., 2011). This view is also supported by mounting genetic evidence in humans with clinically restricted neurological maladies triggered by selective stressors and mutations in the leucine-rich domain of RANBP2 (Neilson et al., 2009; Wolf et al., 2013; Denier et al., 2014). Furthermore, mutations that uncouple selective RBDs of Ranbp2 from Ran GTPase, *Ranbp2* haploinsufficiency or mutations impairing the SUMO-binding motif of Ranbp2 promote neural-type restricted phenotypes in the absence or presence of noxious stressors in mice (Patil et al., 2014; Cho et al., 2010, 2015a).

Several mouse models of MND, such ALS, have been generated, but many of these models rely on the supraphysiological expression of transgenes with disease-causing mutations, because they are predicated on the assumption that MND develops by gain of function of neurotoxic substrates that aggregate in motoneurons (Julien and Kriz, 2006; Peters et al., 2015; Chew et al., 2015; Alexander et al., 2004; Wegorzewska et al., 2009). Notably, substrates and regulators of the Ran GTPase cycle were found to be powerful genetic modifiers of proteotoxicity or proteostasis of ALS substrates (Cho et al., 2015b; Cho et al., 2014; Zhang et al., 2015; Freibaum et al., 2015; Jovičić et al., 2015; Boeynaems et al., 2016). However, therapeutic approaches predicated on supraphysiological mouse models of MND have not produced human therapeutic benefits (Robberecht and Philips, 2013; Turner and Talbot, 2008; Benatar, 2007). These limitations, along with lack of understanding of the physiological roles of regulators of Ran GTPase in MND pathogenesis, highlight the need for novel loss-of-function mouse models of MND that perturb Ran GTPase and its substrates to elucidate the molecular and cellular mechanisms underlying the normal biology and disease processes that occur in motoneurons (Matus et al., 2014). In light of the central role Ranbp2 plays in controlling the Ran GTPase cycle (Patil et al., 2014; Cho et al., 2010; Villa Braslavsky et al., 2000; Vetter et al., 1999; Hamada et al., 2011; Ritterhoff et al., 2016) and the nucleocytoplasmic shuttling and proteostasis of ALS-causing substrates [i.e. hnRNPA2B1 (Kim et al., 2013; Cho et al., 2015b, 2014)], we hypothesized that Ranbp2 plays an instrumental role in motoneuron biology and disease, and tested this idea by producing mouse models lacking Ranbp2 in Thy1 motoneurons of the anterior horns of the spinal cord. We found that loss of Ranbp2 in Thy1 motoneurons of mice causes ALS-like syndromes with hindlimb paralysis, respiratory distress and premature death. These syndromes are caused by multiple physiological disturbances, which include: declines in peripheral nerve conduction velocity and lipid metabolites; profound disruption of nucleocytoplasmic partitioning of Ran GTPase and its substrates, and of Cxc114/Cxc112-Cxcr4-Stat3-mediated chemokine signaling; and paracrine and autocrine neuroglial dysregulation of hnRNPH3 and metalloproteinase 28 (Mmp28) proteostasis.

## RESULTS

### Generation of mice with conditional ablation of *Ranbp2* in Thy1^+^ motoneurons

**Fig. 1.**
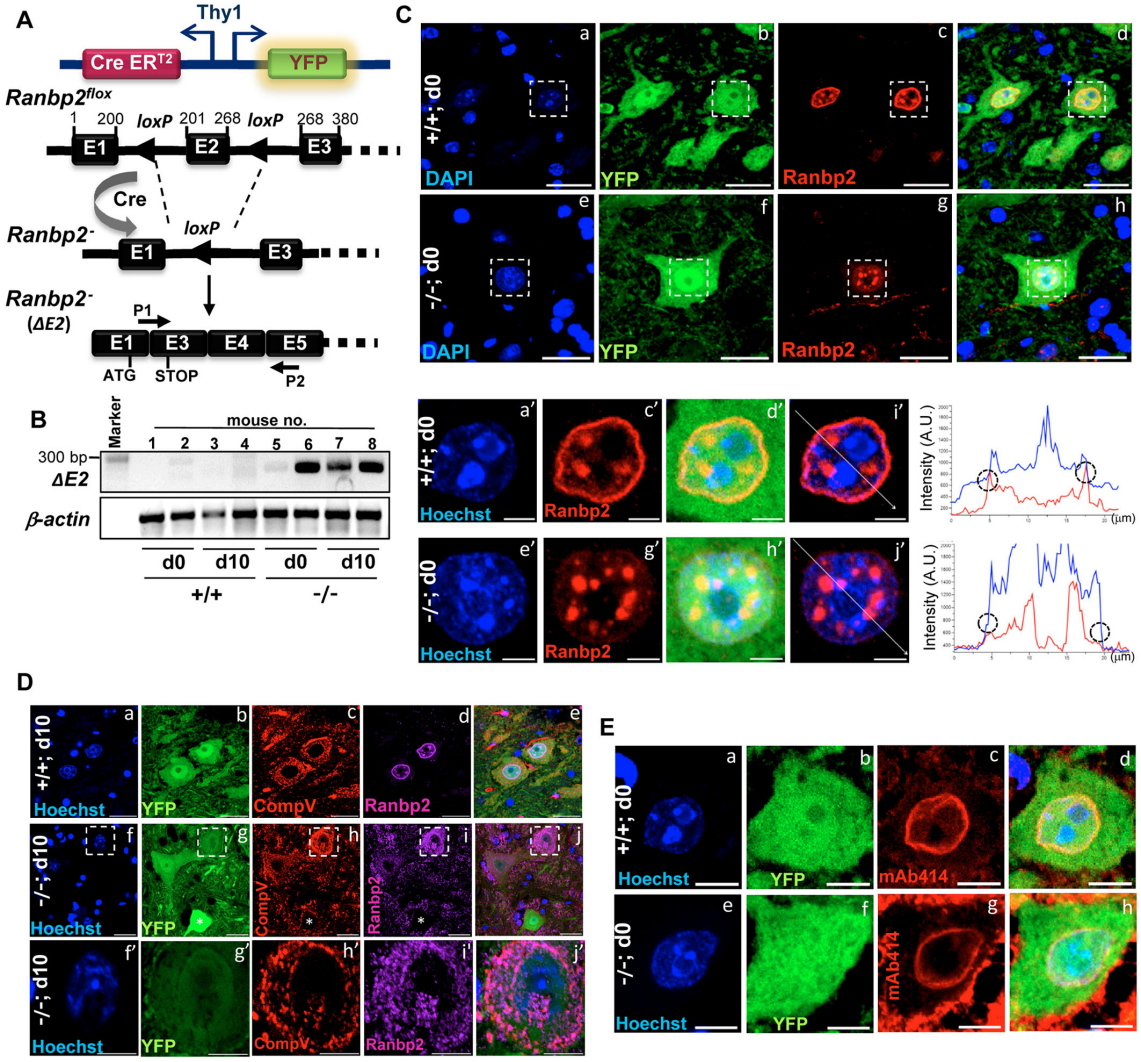
**Genetic ablation of *Ranbp2* in Thy1^+^ motoneurons.** (A) Single-neuron labeling with the inducible Cre-mediated knockout H transgenic line (SLICK-H) co-expressing the yellow fluorescent protein (YFP) and tamoxifen-inducible Cre recombinase (CreER^T2^) under the control of two oppositely oriented *Thy1* promoters was used to excise exon 2 (ΔE2) of *Ranbp2* from mice with a floxed *Ranbp2* gene. P1 and P2 are primers specific for the detection of recombinant (out-of-frame) transcript of Ranbp2; P2 is targeted to the out-of-frame exon 5, whereas P1 comprised the unique recombinant (hybrid) nucleotide sequence produced by the splicing (fusion) of exon 1 with exon 3. Numbering above the exons refers to the nucleotide coordinates of exons. The position of initiation and premature stop codons upon excision of exon 2 are shown. (B) *Ranbp2* mRNA without exon 2 is detected by RT-PCR with P1 and P2 primers in the lumbar spinal cord (L3-L6 level) at the end of a daily 5-day regimen of tamoxifen administration, day 0 (d0), and 10 days (d10) after the last dose of tamoxifen. (C) In comparison with +/+ mice (a-d,a′-i′), YFP^+^-labeled motoneurons of the anterior (ventral) horn of spinal cord (L3-L6) of −/− mice lack Ranbp2 at the nuclear envelope at d0 (e-h,e′-j′). Representative confocal images are equatorial views of nuclei of YFP^+^ neurons. Small and densely Hoechst-stained heterochromatic nuclei of YFP^−^ glia do not express Ranbp2 (a-h). YFP^+^ motoneurons have unique and prominent Ranbp2^+^-intranuclear inclusions (e-h,e′-j′). Images in a′-j′ are magnified views of the insets in a-h and show the line scan (i′,j′) used to plot two-channel fluorescent intensities graphs (right) that show loss of intensity of red channel at the nuclear envelope (circles). (D) At d10, YFP^+^ motoneurons of −/− mice conspicuously lack Ranbp2^+^ intranuclear inclusions; there is prominent miscolocalization of Ranbp2^+^ inclusions with CompV^+^-stained mitochondria. Images in f′-j′ are magnified views of the outlined regions in f-j. (E) The localization of nucleoporins 153 and 62 (Nup153/62) at the nuclear envelope by mAb414 (c,g) is the same in YFP^+^ motoneurons in both −/− and +/+ mice. +/+, *SLICK-H::Ranbp2^+/+^*; −/−, *SLICK-H::Ranbp2^flox/flox^*; CompV, mitochondrial complex V; mAb414, monoclonal Ab414 against Nup358/153/62. Scale bars: 25 µm in Ca-h, Da-j and Ea-h; 5 µm in Ca′-j′ and Df′-j′. d0 and d10 are days 0 and 10 post-tamoxifen administration, respectively. *n>*4 mice/genotype examined (C-E).

To assess the role of Ranbp2 in Thy1^+^ neurons, we genetically excised exon 2 (ΔE2) from the *Ranbp2* floxed gene (Patil et al., 2014; Cho et al., 2013; Dawlaty et al., 2008) by crossing these mice with the single-neuron labeling with inducible Cre-mediated knockout V (*SLICK-V*) or H transgenic lines (*SLICK-H*) lines (Young et al., 2008). These *SLICK* lines co-express the yellow fluorescent protein (YFP) and tamoxifen-inducible Cre recombinase (CreER^T2^) under the control of two oppositely oriented *Thy1* neural-selective promoters that drive the expression of the cell surface glycoprotein Thy1 (*CD90*) and that are differentially expressed among Thy1 neurons of the central and peripheral nervous system of the *SLICK-V* and *SLICK-H* lines (Fig. 1A) (Young et al., 2008). A subsequent study of a *SLICK-H* line also found broader expression of YFP and Cre recombination in the central and peripheral nervous system (Heimer-McGinn and Young, 2011) than that reported by Young et al. (2008), whose observations were closely concordant with our studies.

Excision of exon 2 (ΔE2) from the *Ranbp2* gene produces an out-of-frame *Ranbp2* transcript with fused exons 1 and 3 and a premature termination codon in the out-of-frame exon 3. The encoded protein has only 31 residues instead of the 3053 residues that make up Ranbp2 (Fig. 1A,B, see also Fig. 4A) (Patil et al., 2014; Cho et al., 2013; Dawlaty et al., 2008). Tamoxifen-induced *SLICK-V::Ranbp2^flox/flox^* mice failed to display overt behavioral and physiological phenotypes, despite a loss of Ranbp2 in Thy1 neurons of the central nervous system (CNS) and a lack of overt morphological changes in such neurons upon loss of Ranbp2 expression. By contrast, *SLICK-H::Ranbp2^flox/flox^* mice rapidly developed prominent motor deficits. YFP-neural labeling (and *Ranbp2* ablation) driven by the *Thy1* promoters of *SLICK-V* and *SLICK-H* lines overlap in Thy1 neurons of the CNS (Young et al., 2008), an observation that was concordant with our studies. However, unlike the *SLICK-V* line, the *Thy1* promoters of *SLICK-H* line are broadly expressed in motoneurons of the spinal cord and retinal ganglion neurons (Young et al., 2008). Hence, the focus of this study was on examining the effects of loss of Ranbp2 in Thy1 motoneurons in the anterior horns of the lumbar spinal cord of *SLICK-H::Ranbp2^flox/flox^* mice.

Deletion of ΔE2 of *Ranbp2* was detected in *Ranbp2* mRNA as early as the final day (d0) of a 5-day daily regimen of tamoxifen administration (Fig. 1B). This deletion also led to the typical loss of Ranbp2 at the nuclear rim of YFP^+^ motoneurons in the anterior horns of the lumbar spinal cord (Fig. 1C) and in the central nervous system (Fig. S1). YFP^+^ motoneurons from both *SLICK-H::Ranbp2^+/+^* and *SLICK-H::Ranbp2^flox/flox^* mice also exhibited unique Ranbp2^+^-intranuclear inclusions at day 0 (d0) after the last dose of tamoxifen (Fig. 1C) that have not been previously observed in any other ganglionic or other cell types (Patil et al., 2014; Cho et al., 2014, 2013; Mavlyutov et al., 2002). Although these pre-existing Ranbp2^+^-intranuclear sequestrations were not affected at d0 in *SLICK-H::Ranbp2^flox/flox^* mice, the Ranbp2^+^-intranuclear inclusions were fully mobilized to the cytosolic compartment, where they became colocalized to the mitochondria in YFP^+^ motoneurons of *SLICK-H::Ranbp2^flox/flox^* 10 days (d10) after the last dose of tamoxifen (Fig. 1D). This is reminiscent of the localization of Ranbp2 and some domain constructs thereof to the mitochondrial-rich ellipsoid compartment of photoreceptor neurons and transfected cultured cells, respectively (Cho et al., 2007; Aslanukov et al., 2006). It is possible that the pre-existing and long-lived Ranbp2^+^ intranuclear inclusions make up a novel isoform of Ranbp2, which is distinct from the shorter-lived isoform found at the nuclear rim. We also examined the effect of loss of Ranbp2 at the nuclear envelope on the localization of other nucleoporins, such as Nup62 and Nup153, and found that, as in other studies, the localization of these nucleoporins was not affected (Fig. 1E) (Dawlaty et al., 2008).

### *SLICK-H::Ranbp2^flox/flox^* mice develop severe motor deficits, respiratory distress and premature death

**Fig. 2.**
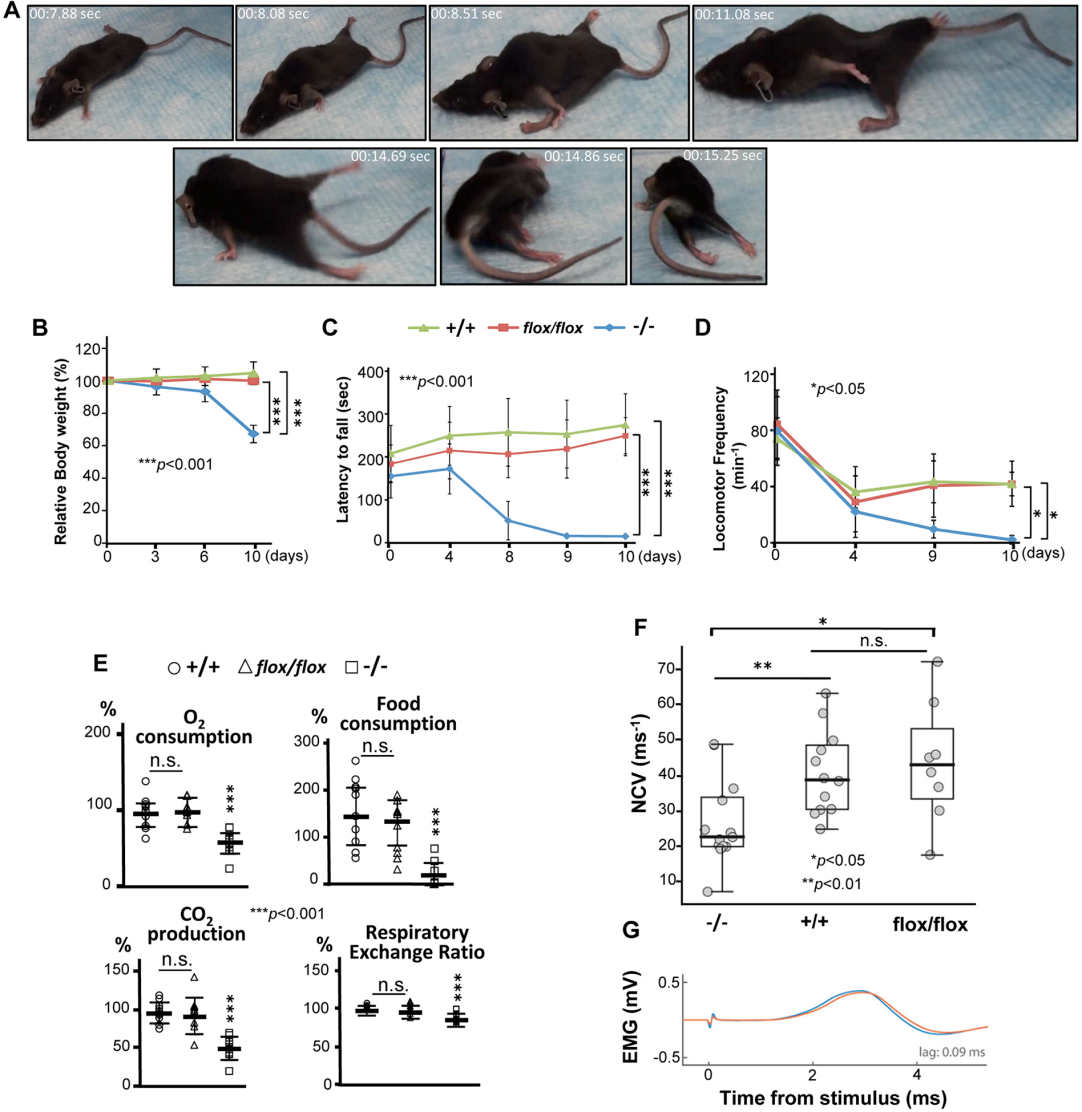
**Decline of motor and metabolic activities of *SLICK-H::Ranbp2^flox/flox^* mice.** (A) Still frames of digital video of locomotor behavior of *SLICK-H::Ranbp2^flox/flox^* (−/−) mice at day 9 post-tamoxifen administration (see also Movie 1). −/− mice develop hind limb paralysis, gait impairment, lack of motor balance and hypoactivity. (B) −/− mice show progressive loss of body weight. (C) Rotarod tests showing strong and progressive loss of motor coordination and balance of −/− mice. (D) Open-field tests showing strong motor hypoactivity of −/− mice at days 9 and 10 post-tamoxifen administration. For behavioral tests (B-D), two-way RMANOVA revealed a significant effect of genotype, *P<0.05, ***P<0.001, *n=*8-10 mice/genotype. Data are expressed as mean±s.d. (E) Oxygen consumption, carbon dioxide production, respiratory exchange ratio and food consumption significantly decline in −/− mice at day 9 post-tamoxifen administration. ***P<0.001, one-way ANOVA, *n=*10 mice/genotype. Data are expressed as mean±s.d. (F) Dot-box plots of sciatic nerve conduction velocity (NCV) adjusted to body temperature. NCV is decreased in −/− mice at day 9 post-tamoxifen administration. Box-plot edges mark the 25th and 75th percentiles of the data, and the centerline shows the median value. **P<0.01, *P<0.05, Kruskal–Wallis test rejected the null hypothesis at *P*=0.014, *n=*8-13 mice/genotype. (G) Representative example of stimulation-evoked electromyography (EMG). The plot shows evoked EMG responses from stimulating the sciatic nerve of a −/− mouse with the electrode nearest to (blue) and farthest from (red) the muscle, and the time lag for which the cross-correlation between the response pair was the largest. Day 0 (d0) is the day after the last dose of a daily 5-day regimen of tamoxifen or vehicle (corn oil) administration; −/−, tamoxifen-treated *SLICK-H::Ranbp2^flox/flox^*; *flox/flox*, vehicle (corn oil)-treated *SLICK-H::Ranbp2^flox/flox^*; +/+, tamoxifen-treated *SLICK-H::Ranbp2^+/+^*.

Loss of Ranbp2 in *SLICK-H::Ranbp2^flox/flox^* mice led to the progressive behavioral phenotypes of gait impairment, lack of motor balance and hypoactivity, hindlimb paralysis and, ultimately, respiratory distress and premature death at day 10.5 (d10.5) (Fig. 2A, Movie 1). By d10, *SLICK-H::Ranbp2^flox/flox^* mice became largely moribund. These traits were accompanied by progressive weight loss between d3 (∼4% of gross weight loss) and d10 (∼33% of gross weight loss) compared with control groups comprising tamoxifen-treated *SLICK-H::Ranbp2^+/+^* and vehicle-treated *SLICK-H::Ranbp2^flox/flox^* mice (*P* values<0.001) (Fig. 2B). These mice were characterized further with behavioral assays. A rotarod test showed that the *SLICK-H::Ranbp2^flox/flox^* mice underwent progressive loss of motor coordination and balance (Fig. 2C). At d8, *SLICK-H::Ranbp2^flox/flox^* displayed poor performance in comparison with two control groups (latency to fall: 44.7±36.8 versus 242.5±78.6 and 191.5±54.8 s for tamoxifen-treated *SLICK-H::Ranbp2^+/+^* and vehicle-treated *SLICK-H::Ranbp2^flox/flox^*, respectively; *P*<0.001) and by d9, *SLICK-H::Ranbp2^flox/flox^* had lost motor coordination and balance (Fig. 2C). *SLICK-H::Ranbp2^flox/flox^* mice also displayed increased hypoactivity in open-field tests that became significantly prominent by d9 compared with two control groups (9.6±6.3 versus 43.2±14.9 and 41.7±8.4 movements min^−1^ for tamoxifen-treated *SLICK-H::Ranbp2^+/+^* and vehicle-treated *SLICK-H::Ranbp2^flox/flox^*, respectively; *P*<0.05) (Fig. 2D). In addition, assays related to food and oxygen consumption, carbon dioxide production and calorimetry showed that *SLICK-H::Ranbp2^flox/flox^* had significant declines in all outcome measures at day 9 (d9) compared with control groups (*P*<0.001) (Fig. 2E). The decline in respiratory exchange ratio (RER) of *SLICK-H::Ranbp2^flox/flox^* is indicative of a metabolic shift towards fat for energy generation (Simonson and DeFronzo, 1990). We also investigated whether the decline in motor activities of *SLICK-H::Ranbp2^flox/flox^* were accompanied by loss of nerve conduction velocity of the sciatic nerve. In comparison with age-matched *SLICK-H::Ranbp2^+/+^* and *SLICK-H::Ranbp2^flox/flox^* mice with and without tamoxifen treatments, respectively, the nerve conduction velocity of *SLICK-H::Ranbp2^flox/flox^* mice was significantly decreased at d9 after the tamoxifen treatment (*P*<0.05) (Fig. 2F,G).

### Morphometric and lipid composition abnormalities in peripheral nerves of *SLICK-H::Ranbp2^flox/flox^* mice

**Fig. 3.**
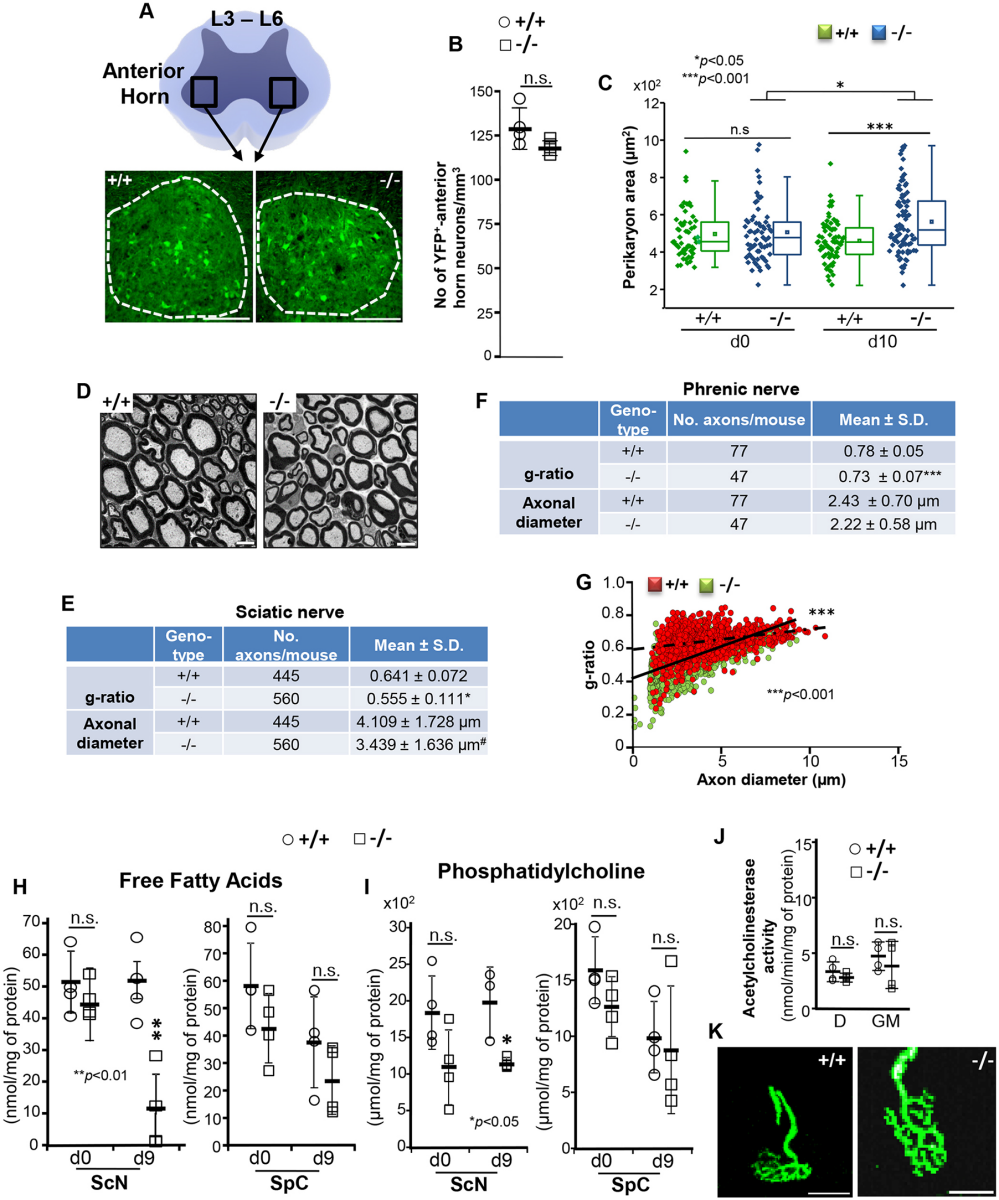
**Morphometric and lipid compositional changes of motoneurons of *SLICK-H::Ranbp2^flox/flox^* mice.** (A) Diagram (top) and representative low-power confocal images (bottom) of the anterior horn at lumbar 3-6 levels (L3-L6) of spinal cords of *SLICK-H::Ranbp2^+/+^* (+/+) and *SLICK-H::Ranbp2^flox/flox^* (−/−) mice. Scale bar: 100 µm. (B) There is no difference in the number of YFP^+^ motoneurons between +/+ and −/− mice. Data are expressed as mean±s.d.; *n=*4 mice/genotype; Student's *t*-tests. (C) Dot and box-plot analyses of perikarya areas of YFP^+^ motoneurons in the anterior horns showing a significant increase of perikarya area (hypertrophy) in −/− mice at d10. Box-plot edges mark the 25th and 75th percentiles of the data, and the centerline shows the median value. ***P<0.001, *P<0.05, Mann–Whitney test, *n=*4 mice/genotype. (D) Transmission electron micrographs of representative ultra-thin cross-sections of sciatic nerves. Scale bar: 5 µm. (E) Quantitative analyses of g-ratios [ratio between the inner (axon) and outer diameter (axon plus myelin sheath) of the myelin sheath] and axonal diameters of sciatic nerves between genotypes. Data are expressed as mean±s.d. ^#^*P*=0.058, *P<0.05, *t*-tests with GEE, *n=*4 mice/genotype. (F) Quantitative analyses of g-ratios and axonal diameters of phrenic nerves between genotypes. Data are expressed as mean±s.d. ***P<0.001, *t*-tests with GEE, *n=*4 mice/genotype. (G) Scatter plots between g-ratio and axonal diameter of fibers of sciatic nerves. Slopes of +/+ and −/− mice are 0.013±0.0017 and 0.038±0.003, respectively; ***P<0.001 (interaction term), *t*-tests with GEE, *n=*4 mice/genotype. Slope values are expressed as mean±s.e.m. (H,I) The sciatic nerve, but not the spinal cord, of −/− mice has decreased levels of free fatty acids (<70%; **P<0.01, Student's *t*-test, *n=*3 or 4 mice/genotype) (H) and phosphatidylcholine (∼50%; *P<0.05, Student's *t*-test, *n=*3 or 4 mice/genotype) (I) compared with +/+ mice at d9. (J,K) The acetylcholinesterase activity in the diaphragm (D) and gastrocnemius muscle (GM) (J), and the structures of neuromuscular junctions (K) appear normal in +/+ and −/− mice. Scale bars: 25 µm. Data are expressed as mean±s.d. in H-J. −/−, *SLICK-H::Ranbp2^flox/flox^*; +/+, *SLICK-H::Ranbp2^+/+^*; d0, d9 and d10 are days 0, 9 and 10 post-tamoxifen administration, respectively.

A decline in nerve conduction velocity often reflects changes between axonal ensheathment and the diameter of peripheral nerves (Waxman, 1980; Rushton, 1951). Hence, we conducted detailed morphometric analyses of multiple parameters within the motoneurons in the anterior horns of the L3-L6 spinal lumbar segments and the sciatic nerve (Fig. 3A). In contrast to other neural and supporting cell types where loss of Ranbp2 causes rampant degeneration (Patil et al., 2014; Cho et al., 2013), we found neither differences in the number of YFP^+^ motoneuron cell bodies (Fig. 3B) nor evidence of apoptotic cell death (TUNEL^+^-cell bodies, data not shown) in *SLICK-H::Ranbp2^+/+^* and *SLICK-H::Ranbp2^flox/flox^* mice; however, there was a significant 1.22-fold increase in the mean perikarya area of YFP^+^ motoneurons of *SLICK-H::Ranbp2^flox/flox^* mice compared with *SLICK-H::Ranbp2^+/+^* motoneurons (*P*<0.001) (Fig. 3C). Nevertheless there were no overt differences in myelination in ultra-thin sections of sciatic nerve between the genotypes (Fig. 3D). Next, we examined morphometric changes in myelination and axonal diameters of sciatic and phrenic nerves. The degree of myelination was quantified by calculating the g-ratios of the sciatic and phrenic nerves, a morphometric parameter that measures changes in the ratio between the diameter of the inner axon alone and that of the axon with myelin sheath. We found that there were significant decreases of ∼15% (*P*<0.05) and 7% (*P*<0.001) in the mean g-ratios, respectively, of sciatic and phrenic nerves between *SLICK-H::Ranbp2^flox/flox^* and *SLICK-H::Ranbp2^+/+^* mice, suggesting that *SLICK-H::Ranbp2^flox/flox^* mice present hypermyelination of these nerves (Fig. 3E,F). As the shrinkage in axon caliber can also mimic the reduced g-ratio typically observed in hypermyelination (O'Neill et al., 1984), we examined also whether the decreases in the g-ratios of sciatic and phrenic nerves from *SLICK-H::Ranbp2^flox/flox^* mice were accompanied by a decline of axonal caliber (Fig. 3E,F). We found that there was a decline, which was of borderline significance, in axon diameters of sciatic nerves of *SLICK-H::Ranbp2^flox/flox^* compared with *SLICK-H::Ranbp2^+/+^* mice (*P*=0.057), whereas there were no significant differences in axon diameters of phrenic nerves between genotypes (*P*=0.10). Thus, these data indicate that an increase in myelination is likely the main reason for this reduced g-ratio. We also examined scatter plots of g-ratios of sciatic fibers as a function of their axonal diameter. This analysis revealed that there was a downward and leftward shift in the scatter plot of *SLICK-H::Ranbp2^flox/flox^* compared with *SLICK-H::Ranbp2^+/+^* mice and that the relationship between axonal diameter and g-ratio (the slope of the regression line) was significantly different for *SLICK-H::Ranbp2^flox/flox^* and *SLICK-H::Ranbp2^+/+^* mice (*P*<0.001) (Fig. 3G). Additional analysis conducted separately for each genotype and nerve also revealed significant slopes for each (*P*<0.001). Similar scatter plot and relationship analyses between axonal diameter and g-ratio (slope) of phrenic nerves also found significant differences for *SLICK-H::Ranbp2^flox/flox^* and *SLICK-H::Ranbp2^+/+^* mice (*P*<0.05). Hence, these data show that there is an increased propensity of a reduced g-ratio for smaller caliber axons (<5 µm) of the sciatic nerves of *SLICK-H::Ranbp2^flox/flox^* mice (Fig. 3G).

We have previously found that haploinsufficiency of *Ranbp2* causes a decrease of free fatty acids in the retina (Cho et al., 2009). The lipid composition is a crucial determinant of the function of nerve fibers (Glynn, 2013; Chrast et al., 2011). In particular, it is thought that declines in fatty acids (FAs) and phosphatidylcholine (PtdCho), which is an abundant source of FA liberation, perturb membrane fluidity, ion permeability and glial energetic support to underlying axons. This decline in FA may also lead to an increase in the capacitance of the sheaths and energy required for membrane depolarization between the nodes. Hence, we examined the levels of free FAs (octanoate and longer fatty acids) (Fig. 3H) and PtdCho (Fig. 3I) in the lumbar spinal cord and sciatic nerves. We found that by d9, sciatic nerves of *SLICK-H::Ranbp2^flox/flox^* mice had significant ∼80 and 50% reductions in the levels of FA (*P*<0.01) and PtdCho (*P*<0.05), respectively, while there were no changes of these lipid metabolites in the spinal cord between genotypes at d0 and d9. Given that motoneurons are cholinergic, we also examined the levels of acetylcholinesterase (AChE) activity in cholinergic synapses of the diaphragm and gastrocnemius muscle (Fig. 3J), and of YFP^+^ nerve terminal structures at neuromuscular junctions (Fig. 3K), and found that there were no differences between genotypes. Microscopic examination of gastrocnemius muscle at d10 found also no signs of denervation atrophy (and muscle wasting) between genotypes (Fig. S2).

### Impairment in subcellular localization of partners and substrates of Ranbp2 in YFP^+^ motoneurons of *SLICK-H::Ranbp2^flox/flo^*^x^ mice

**Fig. 4.**
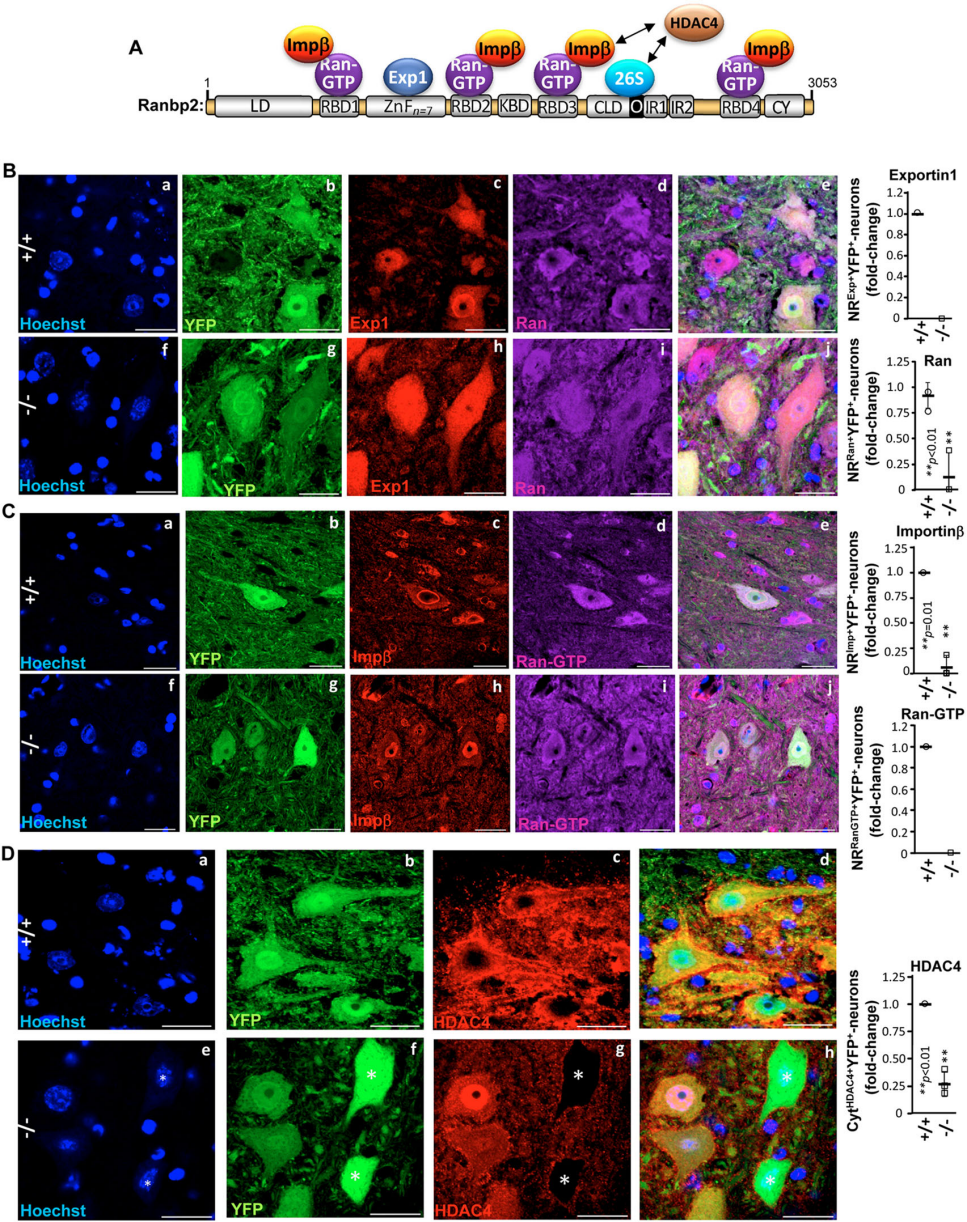
**Subcellular mislocalization of partners and substrates of Ranbp2 in motoneurons of *SLICK-H::Ranbp2^flox/flox^* mice.** (A) Schematic diagram of Ranbp2, its domains and partners thereof. Numbering refers to residues in primary sequence of Ranbp2. Domains are not drawn to scale. (B) Exportin 1 (c,h) is prominently localized at the nuclear rim and excluded from the nucleolus of +/+, whereas there complete loss of nuclear rim staining by exportin 1 and its subcellular distribution becomes highly diffused across all subcellular compartments of YFP^+^ motoneurons of −/− mice. Ran GTPase distribution (d,i) changes from a predominantly cytosolic localization in +/+ to highly diffuse across all subcellular compartments of −/− mice. Ran GTPase localization at the nuclear rim is also strongly decreased among YFP^+^ motoneurons of −/− mice. (C) Importin β (c,h) is prominently localized at the nuclear rim of +/+, whereas its localization at the nuclear rim is strongly decreased among YFP^+^ motoneurons of −/− mice. There is also prominent intranuclear sequestration of importin β in YFP^+^ motoneurons of −/− mice. Ran-GTP localization (d,i) at the nuclear rim is lost among all YFP^+^ motoneurons of −/− mice. (D) HDAC4 subcellular localization (c,g) is drastically changed in −/− mice. HDAC4 is found exclusively in the cytoplasm compartment of all YFP^+^ motoneurons of +/+, but this subcellular partitioning or its expression is lost in YFP^+^ motoneurons of −/− mice. (B-D) Representative images of motoneurons at d10 (day10) post-tamoxifen administration. Scale bars: 25 µm. Graphs on the right in B-D are quantitation analyses of nuclear rim (NR) staining for exportin 1, importin β, Ran GTPase and Ran-GTP, and exclusive cytosolic (Cyt) localization of HDAC4 in YFP^+^ motoneurons of +/+ and −/− mice. Data are expressed as mean±s.d.; *n=*3 or 4 mice/genotype; Student's *t*-tests. LD, leucine-rich domain; RBD*_n_*_=1–4_, Ran GTPase-binding domains, *n*=1-4; ZnF*_n_*_=7_, zinc finger-rich domains; KBD, kinesin 1-binding domain; CLD, cyclophilin-like domain; IR1 and IR2, internal repeats 1 and 2, respectively; M, middle domain between IR1 and IR2; O, overlapping region between CLD and IR1; CY, cyclophilin domain; Exp1, exportin 1; impβ, importin β; HDAC4, histone deacetylase 4; Ran, Ran GTPase; 26S, subunits of the 26S proteasome; −/−, *SLICK-H::Ranbp2^flox/flox^*; +/+, *SLICK-H::Ranbp2^+/+^*.

The multimodular structure of Ranbp2 imparts specific binding and functional activities of domains of Ranbp2 towards selective partners (Fig. 4A). In particular, the Ran-binding domains 1-4 (RBD_1-4_) bind and co-stimulate Ran GTPase activity (Villa Braslavsky et al., 2000; Vetter et al., 1999). The current view is that Ran GTPase activity is required to release importin β and exportin 1 from Ran-GTP, and to initiate and terminate steps of nuclear import and export, respectively, upon the nuclear exit of Ran-GTP•exportin 1 bound to nuclear substrates and Ran-GTP•importin β binary complexes at the cytosolic face of nuclear pores (Cautain et al., 2015). In addition, HDAC4 is a substrate of Ranbp2 whose proteostasis is under cell type-dependent control by Ranbp2 functions that modulate proteasomal and sumoylation activities (Kirsh et al., 2002; Scognamiglio et al., 2008; Knipscheer et al., 2008; Cho et al., 2014). Furthermore, HDAC4 is dysregulated and is a potential therapeutic target in several MNDs (Bruneteau et al., 2013; Bricceno et al., 2012; Li et al., 2012; Williams et al., 2009). Hence, we examined the effects of loss of Ranbp2 on the subcellular partitioning and localization of importin β, exportin 1, Ran-GTP, Ran GTPase and HDAC4 of YFP^+^ motoneurons. In *SLICK-H::Ranbp2^+/+^* mice, exportin 1 (Fig. 4B) and importin β (Fig. 4C) were found prominently at the nuclear rim and excluded from the nucleolus, whereas Ran GTPase (Fig. 4B) and Ran-GTP (Fig. 4C) localizations were largely distributed in the cytoplasmic compartment and excluded from nucleolus. By contrast, there was extensive loss of localization of these Ranbp2 partners at the nuclear rims and changes in their subcellular partitioning in YFP^+^ motoneurons of *SLICK-H::Ranbp2^flox/flox^* mice (*P*≤0.01). For example, exportin 1 (Fig. 4B), Ran GTPase (Fig. 4B) and Ran-GTP (Fig. 4C) were broadly distributed between the cytosolic and nuclear compartments. Furthermore, there was total loss of exportin 1 and Ran-GTP at the nuclear rims of YFP^+^ motoneurons of *SLICK-H::Ranbp2^flox/flox^* mice, whereas Ran GTPase localization was extensively lost at the nuclear rims of YFP^+^ motoneurons (*P*=0.006) (Fig. 4B and C). Importin β was also conspicuously sequestered in the nuclear compartment and its localization at the nuclear rims was lost in most YFP^+^ motoneurons (*P*=0.01) (Fig. 4C). HDAC4 was dispersed throughout foci in the cytoplasmic compartment and excluded from the nucleus of *SLICK-H::Ranbp2^+/+^* mice, whereas HDAC4 was sequestered in the nucleus of YFP^+^ motoneurons or completely absent from the neurons of *SLICK-H::Ranbp2^flox/flox^* mice (*P*=0.002) (Fig. 4D). We also examined whether loss of Ranbp2 promotes changes in subcellular distribution of TDP-43, which is known to relocate from the nuclear to cytosolic compartments of motoneurons in some forms of ALS (Xiao et al., 2015a; Ward et al., 2014). However, TDP-43 was found exclusively in the nucleus of motoneurons, regardless of the genotype (Fig. S3).

### Identification of transcriptional physiological targets of Ranbp2 by differential transcriptome profiling and gene expression analyses

The derangement of nucleocytoplasmic partitioning of nuclear import and export receptors by loss of Ranbp2 is likely to promote transcriptional dysregulation by impairing the nuclear import of selective transcriptional factors and/or nuclear export of transcripts in motoneurons. To gain insights into the role of Ranbp2 in transcriptional regulation of motoneurons, we conducted differential transcriptome profiling by deep RNA sequencing (RNAseq) (Wang et al., 2009) of transcriptomes of the sciatic nerve. Transcriptome profiling with a sequence depth between 45-70 million seq reads were performed on *SLICK-H::Ranbp2^flox/flox^* and *SLICK-H::Ranbp2^+/+^* mice, and on a transgenic line, Tg^RBD2/3*-HA^ ::*SLICK-H::Ranbp2^flox/flox^*, which expresses Ranbp2 with loss of function of its RBD2 and RBD3 (RBD2/3) (Patil et al., 2014) in motoneurons with a null *Ranbp2* background. The rationale to include the Tg^RBD2/3*-HA^::*SLICK-H::Ranbp2^flox/flox^* line and the sciatic nerve as biological sources in differential transcriptome analyses between *SLICK-H* genotypes was due to several reasons. First, genetic complementation studies in mice with BAC *Ranbp2* constructs harboring loss-of-function mutations in selective domains of Ranbp2 (e.g. RBD2/3) expressed in motoneurons with a null *Ranbp2* background completely rescued the behavioral defects displayed by *SLICK-H::Ranbp2^flox/flox^* mice (Patil et al., 2014; Cho et al., 2014). Second, although the activities of RBD2/3 of Ranbp2 are selectively vital to other cell types (Patil et al., 2014), the Tg^RBD2/3*-HA^::*SLICK-H::Ranbp2^flox/flox^* line serves as an additional control to filter out transcriptomal changes that are physiologically and specifically dispensable to motoneuronal function. Finally, the simple (low) cellular heterogeneity of the sciatic nerve, which is made up mostly of Schwann cells and motoneuron axons, mitigates the interference of transcriptional noise contributed by diverse and genetically unmodified cell types that typically make up other neuroglial systems of higher complexity, such as the spinal cord.

**Fig. 5.**
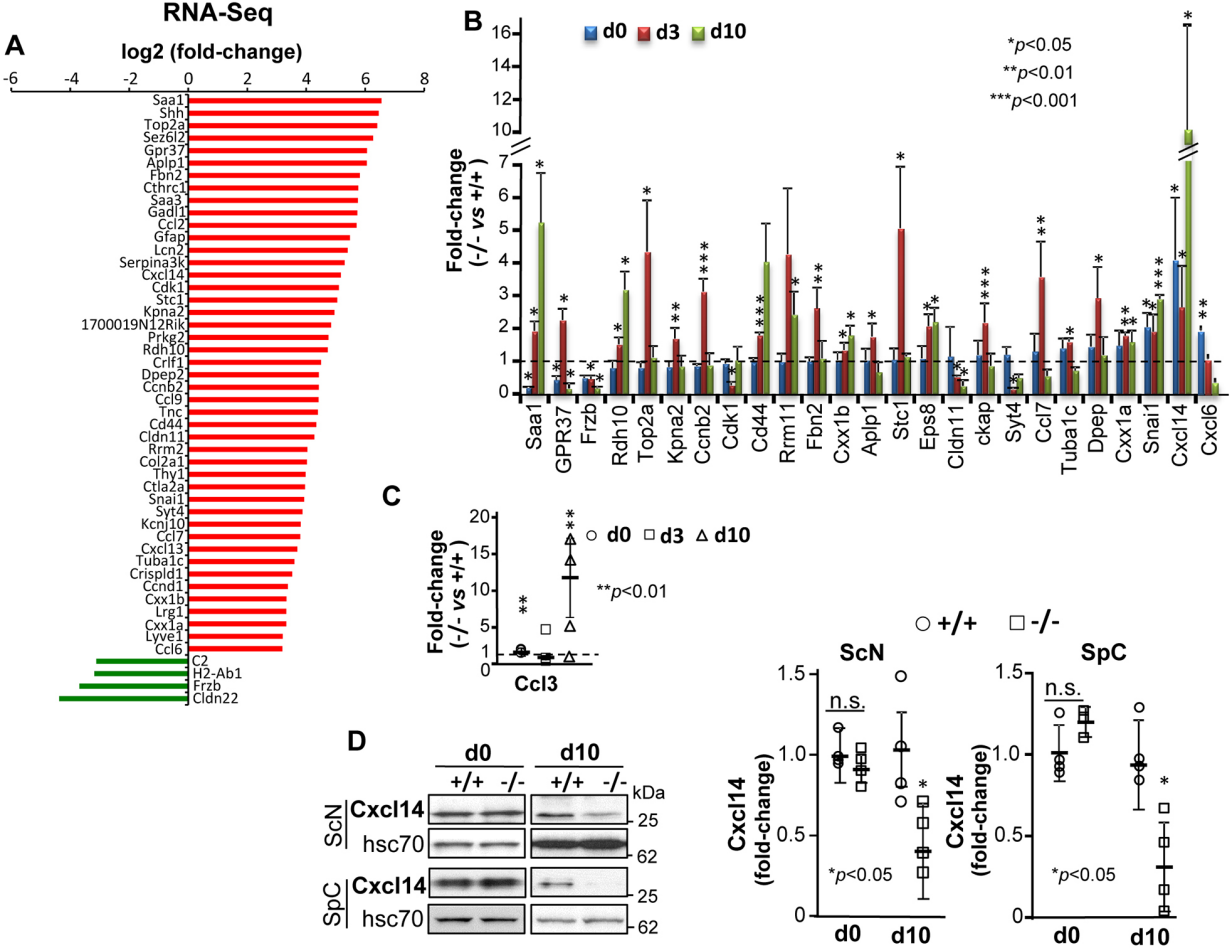
**Differential transcriptome and gene expression analyses of sciatic nerve upon loss of Ranbp2 in motoneurons.** (A) Differential RNA-Seq-based whole-transcriptome analysis of sciatic nerves between *SLICK-H::Ranbp2^flox/flox^* (−/−), *SLICK-H::Ranbp2^+/+^* (+/+) and Tg^RBD2/3*-HA^::SLICK-H::Ranbp2^flox/flox^ (Tg^RBD2/3*-HA^::−/−) mice. Forty-five upregulated and four downregulated transcripts were found in *−/−* mice at day 10 post-tamoxifen administration. (B) Validation of ranked RNA-Seq dataset by RT-qPCR and temporal and directional changes of expression of mRNAs between −/− and +/+ mice. Twenty-four transcripts and *Frzb* were confirmed to be uniquely upregulated and downregulated, respectively, by RT-qPCR in *−/−* sciatic nerve. *Cxcl14* had the strongest upregulation (∼10.5-fold) as early as day 3 (d3). *P<0.05, **P<0.01, ***P<0.001, Student's *t*-test, *n*=3 or 4 mice/genotype. (C) Temporal and transcriptional changes in expression of *Ccl3* in sciatic nerves at d0, d3 and d10. **P<0.01, Student's *t*-test, *n*=3 or 4 mice/genotype. (D) Immunoblots (left) and quantification of Cxcl14 (right) from equal amounts of homogenates of sciatic nerves (ScN) and spinal cords (SpC) at d0 and d10. Cxcl14 levels are decreased in ScN and SpC at d10. *P<0.05, Student's *t*-test, *n*=4 mice/genotype. Hsc70 is a loading control. All data are expressed as mean±s.d. −/−, *SLICK-H::Ranbp2^flox/flox^*; +/+, *SLICK-H::Ranbp2^+/+^*; hsc70, heat shock protein 70; d0 and d10 are days 0 and 10 post-tamoxifen administration, respectively.

Using differential transcriptome profiling between *SLICK-H::Ranbp2^flox/flox^* and *SLICK-H::Ranbp2^+/+^* sciatic nerves at d10, and after filtering out transcriptional changes shared by Tg^RBD2/3*-HA^::*SLICK-H::Ranbp2^flox/flox^* sciatic nerves, we identified 45 upregulated transcripts and four downregulated transcripts with a log_2_ fold-change (FC)≥ |4| cutoff and false discovery rate (FDR)<0.05 (*q*<0.05) in *SLICK-H::Ranbp2^flox/flox^* mice of sciatic nerves at d10 (Fig. 5A). We then independently validated the direction, relative magnitude and temporal changes of RNA-Seq transcripts by qRT-PCR at d0, d3 and d10. Twenty-five differentially expressed transcripts (DETs) were validated at specific or all time points. Twenty-four DETs were upregulated, while *Frzb* was downregulated (Fig. 5B). The top two upregulated DETs were the chemokine (C-X-C motif) ligand 14 (*Cxcl14*, 10-fold; *P* values<0.05) and serum amyloid A1 (*Saa1*, 5-fold; *P* values<0.05); the changes in transcriptional levels occurred as early as d0 and progressively increased until d10 post-tamoxifen treatment (Fig. 5B). By contrast, there was a significant 10-fold decrease in the levels of secreted frizzle-related protein 3 precursor (Sfrp3/*Frzb*) by d3 in *SLICK-H::Ranbp2^flox/flox^* mice (*P*<0.05) (Fig. 5B). Some transcriptional changes were also transient and they likely reflect selective responses to pathophysiological stages of motoneuron disease. We expanded the longitudinal qRT-PCR analysis to another chemokine ligand, *Ccl3*, as other chemokine (C-C motif) ligands with potential overlapping or complementary functions were found to be upregulated by RNA-Seq and qRT-PCR at different time points (e.g. Ccl7 and Ccl6) (Anders et al., 2014). As shown in Fig. 5C, *Ccl3* was significantly upregulated at d0 and d10 in *SLICK-H::Ranbp2^flox/flox^* compared with *SLICK-H::Ranbp2^+/+^* mice (*P* values<0.01). In light of the robust increase in the levels of *Cxcl14* among all upregulated chemokines, we examined the steady-state translational levels of Cxcl14 in the spinal cord and sciatic nerve between genotypes. Cxcl14 levels were unchanged between genotypes at d0 in the spinal cord and sciatic nerve, but by d10, there was a paradoxical halving or more of Cxcl14 levels in both the spinal cord and sciatic nerve of *SLICK-H::Ranbp2^flox/flox^* compared with *SLICK-H::Ranbp2^+/+^* mice (*P* values<0.05) (Fig. 5D) that contrasts with the strong transcriptional upregulation of *Cxcl14* found between these genotypes of mice (Fig. 5A,B).

### Subcellular sequestration of chemokine signaling components in motoneurons of *SLICK-H::Ranbp2^flox/flox^*

**Fig. 6.**
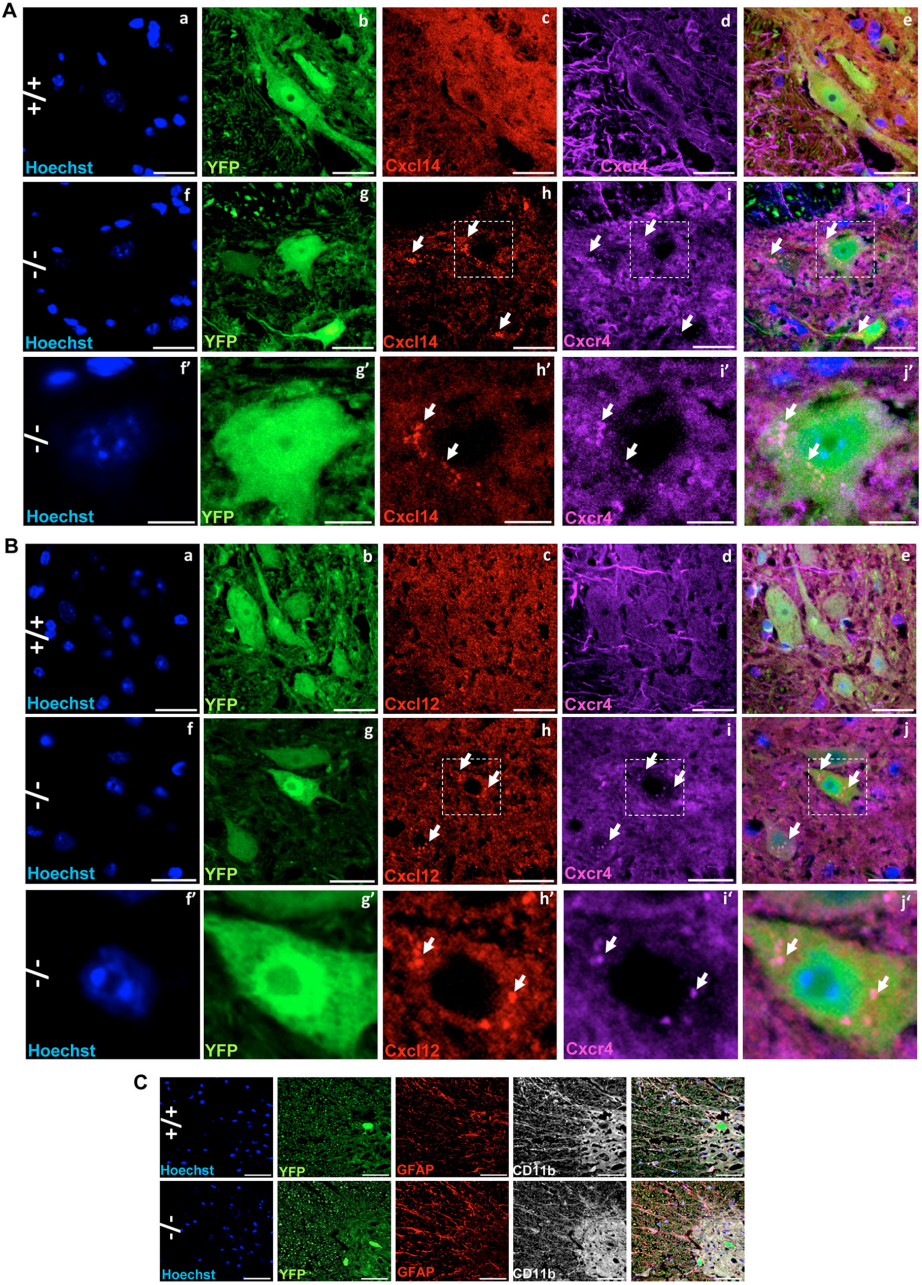
**Intracellular sequestration and aggregation of the chemokine receptor Cxcr4 and its ligands, Cxcl14 and Cxcl12, in motoneurons of *SLICK-H::Ranbp2^flox/flox^* mice.** (A,B) Representative images of Cxcr4, Cxcl14 and Cxcl12 subcellular localizations in YFP^+^ motoneurons in different genotypes. +/+ mice have weak immunostaining and a diffuse cellular distribution of Cxcr4 and Cxcl14 (A,a-e) and Cxcl12 (B,a-e) in YFP^+^ motoneurons of the anterior horn, whereas there is intracellular colocalization, sequestration and aggregation of Cxcr4 with Cxcl14 (A,f-j) and Cxcl12 (B,f-j) in YFP^+^ motoneurons of −/− mice. (f′-j′) Magnified images of the outlined regions indicated in f-j. (C) There are no signs of overt glyosis and microglia activation in the anterior horns at d10 (day 10) post-tamoxifen administration, as shown by the absence of GFAP^+^ and Cd11b^+^ immunostaining between genotypes. Scale bars: 25 µm. −/−, *SLICK-H::Ranbp2^flox/flox^*; +/+, *SLICK-H::Ranbp2^+/+^*. Arrows indicate intracellular aggregate or deposit foci.

**Fig. 7.**
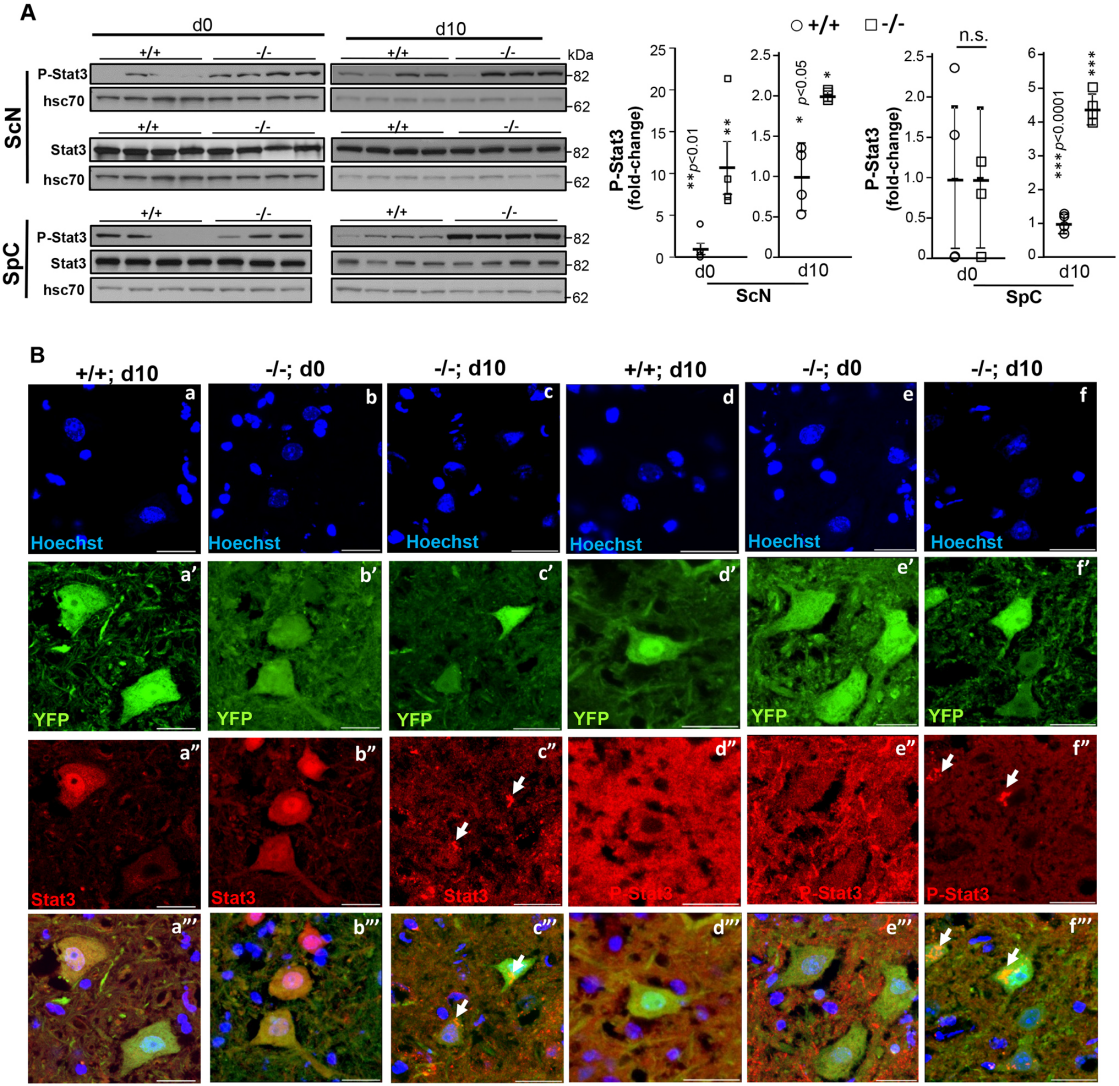
**Loss of Ranbp2 in motoneurons causes the accumulation and intracellular aggregation of latent and activated Stat3.** (A) Immunoblots of activated Stat3 (P-Stat3) show that loss of Ranbp2 in motoneurons causes a pronounced accumulation of activated Stat3 in the sciatic nerve (ScN; >10-fold at d0) and spinal cord (SpC; >4-fold at d10) as early as d0 and at d10, respectively. **P<0.01, ***P<0.001, Student's *t*-test, *n*=4 mice/genotype. Representative immunoblots (left) of equal amounts of tissue homogenates and quantitative analyses of P-Stat3 (graph, right) are shown. Hsc70 is a loading control. For ScN d0, the same blots were used for P-Stat3, Stat3, Mmp28 and hsc70 in Figs 7 and 9. For ScN d10, the same blot was used for P-Stat3, Stat3 and hsc70. For SpC d0, the same blots were used for P-Stat3, Stat3, hnRNPH3, Mmp28 and hsc70 in Figs 7-9. For SpCd10, same blots were used for P-Stat3, Stat3 and Mmp28 and hsc70 in Figs 7 and 9. A statistical outlier was excluded in the dataset of P-Stat3 in ScN at d10. Lanes shown are experimental replicates. Data are expressed as mean±s.d. (B) Representative confocal images of anterior horn immunostained for latent and activated Stat3. There is a prominent aggregation of latent and activated Stat3 in −/− motoneurons at d10. There is also loss of subcellular partitioning of P-Stat3 between the nuclear and cytosolic compartments of −/− motoneurons at d0 and an aggregation of P-Stat3 at d10. −/−, *SLICK-H::Ranbp2^flox/flox^*; +/+, *SLICK-H::Ranbp2^+/++/+^*; hsc70, heat-shock protein 70; d0 and d10 are days 0 and 10 post-tamoxifen administration, respectively. Arrows indicate intracellular aggregate or deposit foci.

Given that Cxcl14 may act as a natural inhibitor ligand of Cxcr4 (Tanegashima et al., 2013; Hara and Tanegashima, 2014), which is a member of the subfamily A2 of rhodopsin-like G protein-coupled receptors (Wolf and Grunewald, 2015), and that its binding to Cxcr4 may influence Cxcl14-Cxcr4 subcellular localization, we examined the subcellular distribution of Cxcl14 and Cxcr4 in YFP^+^ motoneurons of the anterior horns. We also extended this analysis to Cxcl12 and Stat3, because Cxcl12 is another ligand of Cxcr4, its binding to Cxcr4 activates Stat3 (Vila-Coro et al., 1999; Ahr et al., 2005), and Ranbp2 [via its cyclophilin (CY) domain] associates with latent and activated Stat3 (Cho et al., 2014) and modulates the *trans*-activation potential of Stat3 in gene expression (Cho et al., 2015b). Examination of the intracellular localization of Cxcr4 signaling components in YFP^+^ motoneurons found that compared with *SLICK-H::Ranbp2^+/+^* mice, there was widespread aggregation and colocalization of Cxcr4, Cxcl12 and Cxcl14 at conspicuous intracellular foci of YFP^+^ motoneurons of *SLICK-H::Ranbp2^flox/flox^* mice (Fig. 6A,B). These Cxcr4^+^Cxcl12^+^ and Cxcr4^+^Cxcl14^+^ foci were found typically at the perinuclear region of YFP^+^ motoneurons (arrows, Fig. 6A,B). As Cxcr4, Cxcl12 and Cxcl14 are implicated in pro-inflammatory signaling, we also examined whether gliosis was elicited in the anterior horn of *SLICK-H::Ranbp2^flox/flox^* mice. As shown in Fig. 6C, we found no evidence of paracrine activation of GFAP^+^ macroglia or Cd11b^+^ microglia in the anterior horn. Likewise, we did not find any CD45^+^ microglia (not shown). Next, we examined the effects of loss of Ranbp2 in Stat3 proteostasis in the spinal cord and sciatic nerve. In comparison with *SLICK-H::Ranbp2^+/+^*, *SLICK-H::Ranbp2^flox/flox^* mice had a pronounced upregulation of activated Stat3 (P-Stat3) in the sciatic nerve as early as d0 (*P* values<0.01), whereas activated Stat3 (P-Stat3) was significantly upregulated in the spinal cord by d10 (*P<*0.001) (Fig. 7A). In YFP^+^ motoneurons of the spinal cord, there were prominent aggregation foci of latent and activated Stat3 in the cytoplasm compartment of YFP^+^ motoneurons of *SLICK-H::Ranbp2^flox/flox^* mice at d10, whereas such Stat3 foci were absent in age-matched *SLICK-H::Ranbp2^+/+^* mice (Fig. 7B).

### Post-transcriptional dysregulation of hnRNPH3 in *SLICK-H::Ranbp2^flox/flox^*

**Fig. 8.**
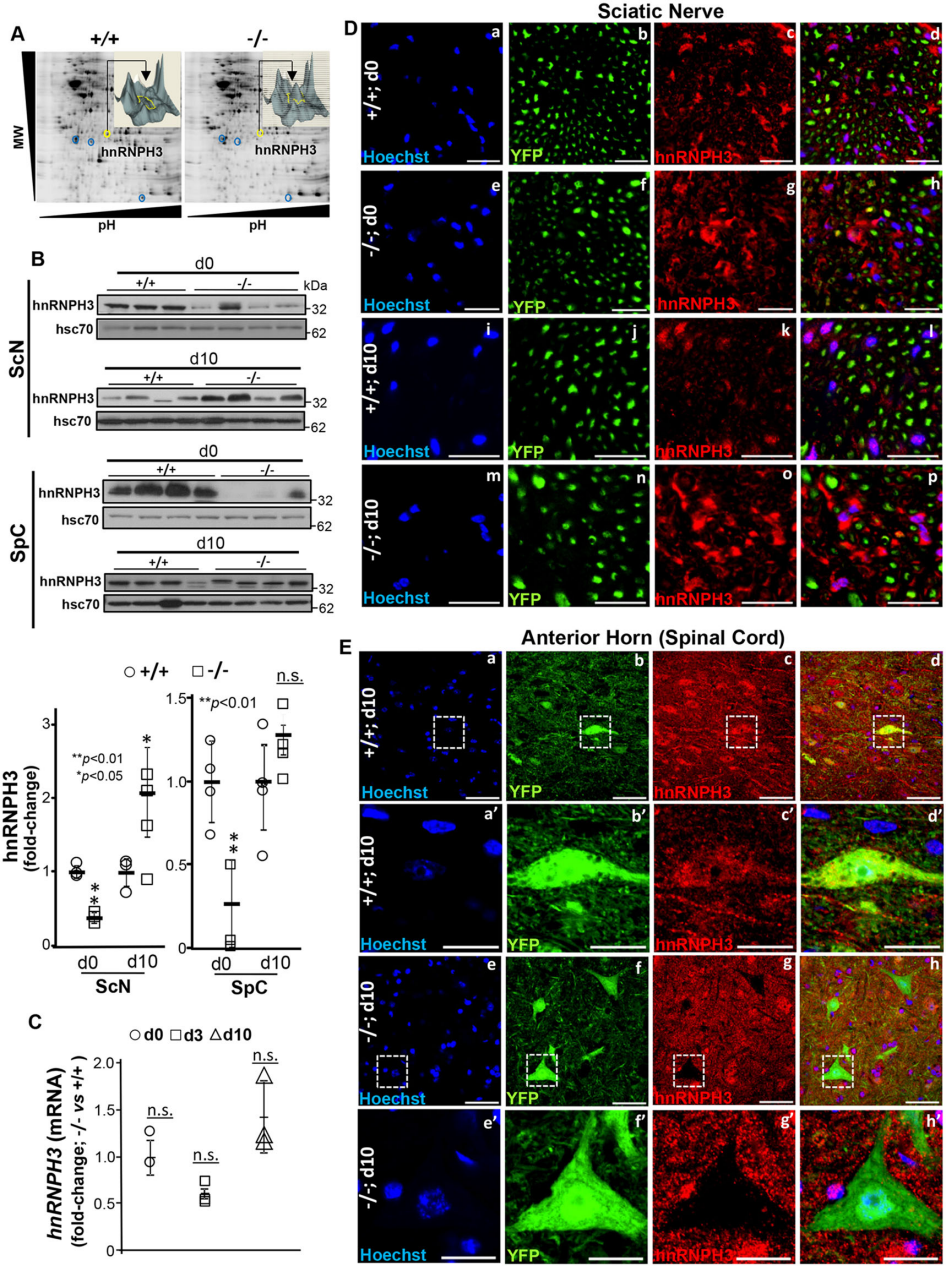
**Post-transcriptional deregulation of hnRNPH3 in sciatic nerve and neural cell bodies by loss of Ranbp2.** (A) Proteomic analyses by 2D-DIGE of homogenates of optic nerves of +/+ and −/− mice at d10. Insets are volumetric analyses of spot intensities marked by the yellow circle and arrow and that show an increase of a protein, which was identified by mass spectrometry as hnRNPH3, between +/+ and −/− mice. (B) Immunoblots (top panel) of equal amounts of homogenates and quantitative analyses (bottom panel) showing dysregulation of hnRNPH3 proteostasis in sciatic nerve (ScN) and spinal cord (SpC) at d0 and d10. *P<0.05, **P<0.01, Student's *t*-test, *n*=4 mice/genotype. Data are expressed as mean±s.d. Hsc70 is a loading control. For SpC d0, the same blots were used for P-Stat3, Stat3, hnRNPH3, Mmp28 and hsc70 in Figs 7-9. A statistical outlier was excluded in the dataset of hnRNPH3 in ScN at d0. Lanes shown are experimental replicates. (C) RT-qPCR showing that the temporal and transcriptional profile of *hnRNPH3* mRNA in the sciatic nerve is not changed between +/+ and −/− mice. Student's *t*-test, *n=*4 mice/genotype. Data are expressed as mean±s.d. (D,E) Representative confocal images of cross-sections of sciatic nerve (D) and spinal cord (E) immunostained for hnRNPH3. (D) YFP^+^Hoechst^−^-axons lack hnRNPH3 and hnRNPH3 is localized in YFP^−^ Hoechst^+^-Schwann cells, which have enhanced immunostaining of hnRNPH3 in −/− mice at d10. (E) The soma of  YFP^+^ motoneurons in −/− anterior horns conspicuously lack hnRNPH3. Images of a′-d′ and e′-h′ are magnified views of the outlined regions in a-d and e-h, respectively. −/−, *SLICK-H::Ranbp2^flox/flox^*; +/+, *SLICK-H::Ranbp2^+/++/+^*; hsc70, heat shock protein 70; d0 and d10 are days 0 and 10 post-tamoxifen administration, respectively. Scale bars*:* 50 µm in Da-p,Ea-h; 20 µm in Ea′-h′; n.s., non-significant.

Loss of peptidyl prolyl-isomerase activity of the cyclophilin domain (CY) in Ranbp2 promotes the downregulation of the proteostasis of the ALS-causing substrate, hnRNPA2B1, in inner retinal neurons (Cho et al., 2014), whereas orthosteric inhibitors of the peptidyl prolyl-isomerase activity of CY of Ranbp2 downregulate hnRNPA2B1 proteostasis (Cho et al., 2015b). Although we found that hnRNPA2B1 was extremely abundant in the spinal cord and sciatic nerves, there was no evidence for changes in hnRNPA2B1proteostasis in these tissues between *SLICK-H::Ranbp2^+/+^* and *SLICK-H::Ranbp2^flox/flox^* mice (data nor shown). Hence, we took an unbiased and independent proteomic approach to evaluate proteostatic changes in sciatic and optic nerves between *SLICK-H::Ranbp2^+/+^* and those of *SLICK-H::Ranbp2^flox/flox^* and Tg^RBD2/3*-HA^::*SLICK-H::Ranbp2^flox/flox^* mice, by employing two-dimensional difference in-gel electrophoresis (2D-DIGE) analysis of homogenates of the nerves of these mice. This 2D-DIGE approach led to the isolation of ∼22 proteins whose levels were changed by at least twofold between genotypes. Among these proteins, however, only a single protein with a significant change of expression between genotypes could be validated by independent approaches. As shown in Fig. 8A, we identified a protein, the level of which was increased in *SLICK-H::Ranbp2^flox/flox^* mice by d10 compared with age-matched *SLICK-H::Ranbp2^+/+^* mice (and Tg^RBD2/3*-HA^::*SLICK-H::Ranbp2^flox/flox^*, data not shown). Tandem mass spectrometry identified the protein to be heterogeneous nuclear ribonucleoprotein H3 (hnRNPH3). hnRNPH3 identity and the relative levels between genotypes were independently validated by immunoblot analysis. hnRNPH3 was significantly increased in *SLICK-H::Ranbp2^flox/flox^* sciatic nerve by d10 (*P*<0.05), but it was significantly decreased at d0 in the sciatic nerve and spinal cord (*P* values<0.01) (Fig. 8B). These proteostatic changes in hnRNPH3 occurred without alterations in its transcriptional steady-state levels (Fig. 8C). Next, we examined whether changes in hnRNPH3 proteostasis were reflected in its subcellular distribution in the sciatic nerve and YFP^+^ motoneurons between genotypes. The hnRNPH3 was restricted to Schwann cells surrounding YFP^+^-axons of motoneurons and *SLICK-H::Ranbp2^flox/flox^* sciatic nerves appeared to accumulate hnRNPH3 by d10 (Fig. 8D). Paradoxically, and compared with soma of YFP^+^ motoneurons of *SLICK-H::Ranbp2^+/+^* mice, the soma of YFP^+^ motoneurons of *SLICK-H::Ranbp2^flox/flox^* anterior horns completely lacked hnRNPH3 immunostaining at d10 (Fig. 8E).

### *SLICK-H::Ranbp2^flox/flox^* harbor post-transcriptional deregulation of Mmp28

Loss of Ranbp2 in other cell types, such a photoreceptor neurons and retinal pigment epithelium, promotes the selective and early upregulation and activation of metalloproteinase 11 (Mmp11), and its secretion to the interstitial space, where it exerts crucial non-autonomous cellular and pathogenic effects on neighboring healthy neurons (Patil et al., 2014; Cho et al., 2013). However, we found that Mmp11 was not upregulated in the spinal cord and sciatic nerve of *SLICK-H::Ranbp2^flox/flox^* mice (data not shown). Hence, we extended our analysis to another metalloproteinase, Mmp28, because this metalloproteinase, like Mmp11, is activated in the intracellular secretory pathway (Illman et al., 2003) and several Mmps are implicated in chemokine processing (Van Lint and Libert, 2007). Furthermore, Mmp28 was found to modulate myelination in the peripheral nervous system and to be upregulated in neurological disease conditions, such as experimental autoimmune encephalitis and multiple sclerosis (Werner et al., 2008), where it may act as a negative regulator of macrophage chemotaxis by mechanisms that are elusive (Gharib et al., 2014).

**Fig. 9.**
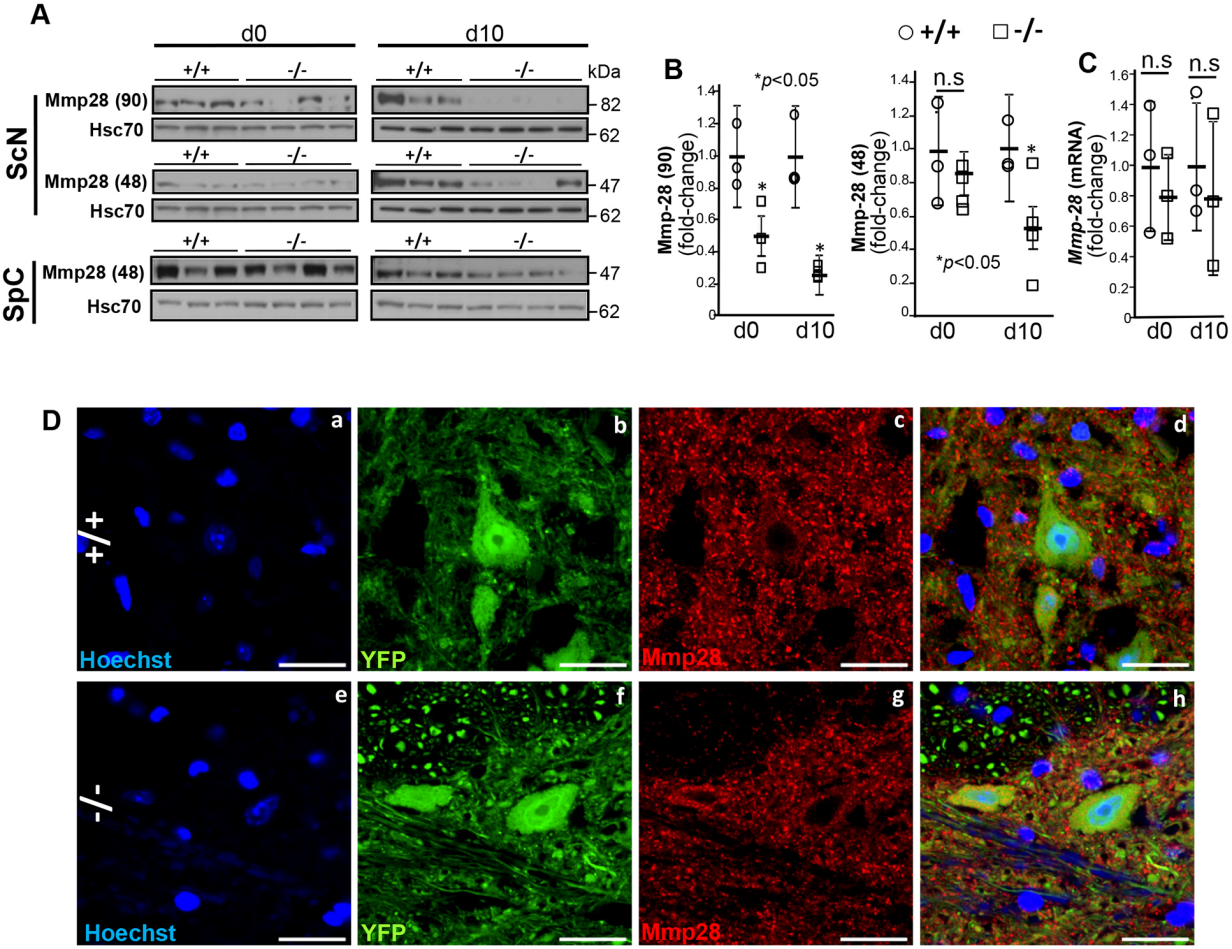
**Post-transcriptional dysregulation of Mmp28 in the sciatic nerve.** (A) Mmp28 resolves in SDS-PAGE as two distinct electrophoretic isoforms with apparent masses of 48 [Mmp28 (48)] and 90 kDa [Mmp28 (90)]. Immunoblots of equal amounts of homogenates of sciatic nerve (ScN) and spinal cord (SpC) show that Mmp28 (90) and Mmp28 (48) levels are reduced only in −/− sciatic nerve. Hsc70 is a loading control. For ScN d0, the same blots were used for P-Stat3, Stat3, Mmp28 and hsc70 in Figs 7 and 9. For SpC d0, the same blots were used for P-Stat3, Stat3, hnRNPH3, Mmp28 and hsc70 in Figs 7-9. For SpCd10, the same blots were used for P-Stat3, Stat3, Mmp28 and hsc70 in Figs 7 and 9. Lanes shown are experimental replicates. (B) Quantification analyses of immunoblots of Mmp28 in ScN showing a significant decrease in the levels of Mmp28 (90) at d0 and d10, and of Mmp28 (48) at d10 in −/− mice. *P<0.05, Student's *t*-test, *n*=4 mice/genotype. Data are expressed as mean±s.d. (C) RT-qPCR showing that the temporal and transcriptional profile of *Mmp28* mRNA in the sciatic nerve is not changed between +/+ and −/− mice. Student's *t*-test, *n*=4 mice/genotype. Data are expressed as mean±s.d. (D) Representative confocal images of motoneurons in the anterior horn immunostained for Mmp28 show that YFP^+^ motoneurons do not show changes in the Mmp28 subcellular localization between genotypes. −/−, *SLICK-H::Ranbp2^flox/flox^*; +/+, *SLICK-H::Ranbp2^+/++/+^*; hsc70, heat shock protein 70; d0 and d10 are days 0 and 10 post-tamoxifen administration, respectively. Scale bars: 25 µm; n.s., non-significant.

We found that Mmp28 in the sciatic nerve resolved in SDS-PAGE as two isoforms of 48 [Mmp28 (48)] and 90 kDa [Mmp28 (90)], whereas Mmp28 (48) was found only in the spinal cord (Fig. 9A-B). The Mmp28 (90) isoform likely represents a SDS-resistant dimer or a post-translational modified isoform of Mmp28 (48). In sciatic nerves of *SLICK-H::Ranbp2^flox/flox^* mice, Mmp28 (48) and Mmp28 (90) were significantly downregulated by over twofold compared with *SLICK-H::Ranbp2^+/+^* mice (*P* values<0.05) (Fig. 9A,B). By contrast, we did not observe changes in Mmp28 proteostasis in the spinal cord between genotypes (Fig. 9A). Furthermore, the changes of Mmp28 proteostasis in *SLICK-H::Ranbp2^flox/flox^* sciatic nerves were accompanied neither by changes in the transcriptional levels of Mmp28 at d0 and d10 (Fig. 9C), nor by overt alterations in the subcellular distribution of Mmp28 in spinal YFP^+^ motoneurons between genotypes (Fig. 9D). As noted previously, Ranbp2 modulates the activity of the ubiquitin proteasome system (UPS) in photoreceptor neurons and the UPS controls the activity and proteostasis of selective substrates of Ranbp2 (Cho et al., 2014, 2010; Yi et al., 2007; Scognamiglio et al., 2008). Hence, we measured the total levels of ubiquitin (free ubiquitin and ubiquitylated proteins) in sciatic nerves to ascertain whether changes in proteostasis of Mmp28 (and hnRNPH3) were caused by overall changes in the UPS activity. However, we found that the levels of total ubiqutin in the sciatic nerve of motoneurons were similar between genotypes (Fig. S4).

## DISCUSSION

**Fig. 10.**
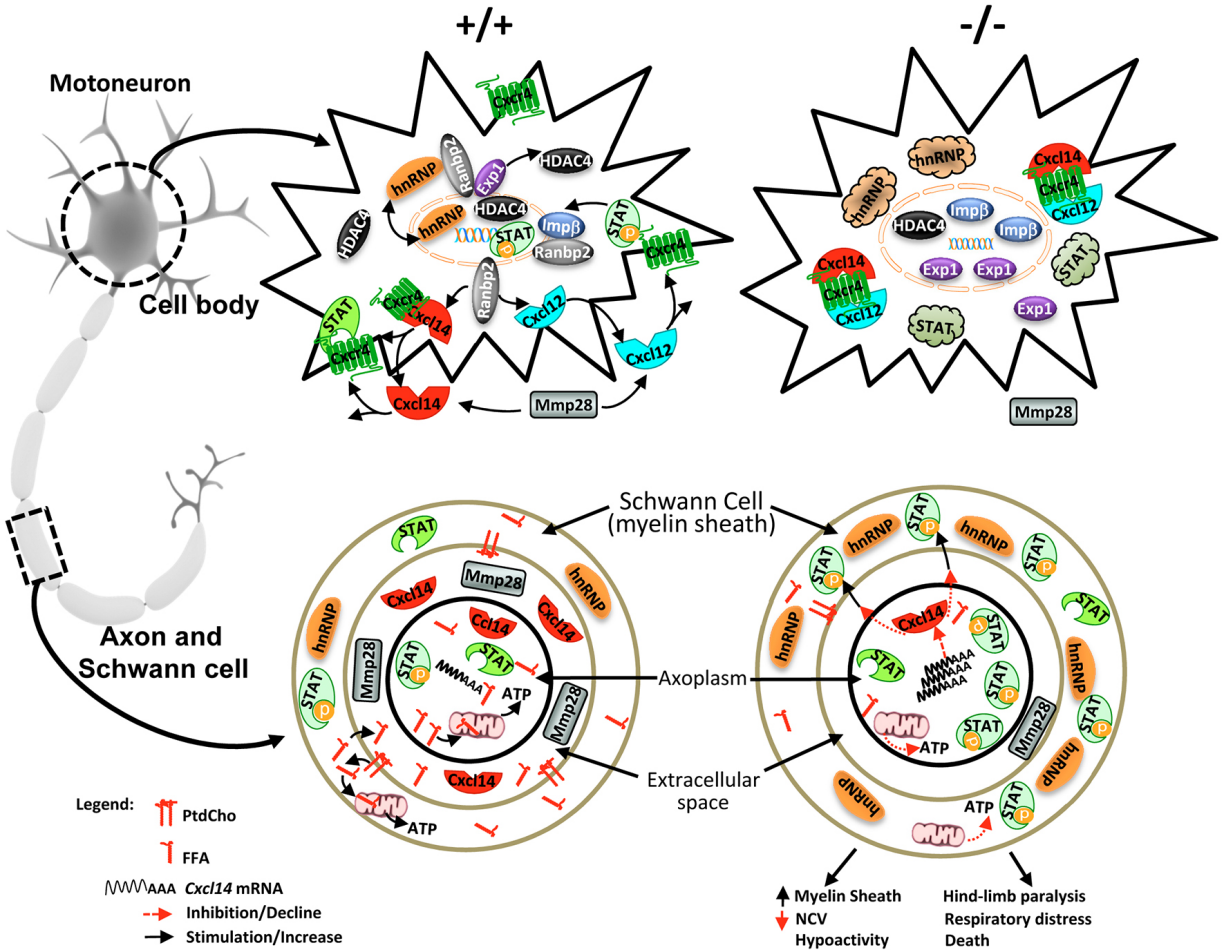
**Mechanistic model of Ranbp2 functions in motoneuron and disease.** In cell bodies of motoneurons of wild-type mice (upper left), Ranbp2 localizes at the cytosolic face of the nuclear pore complex, where it couples with the nuclear import and export receptors importin β and exportin 1, respectively, and controls rate-limiting steps of nucleocytoplasmic shuttling of these receptors and substrates, such as HDAC4, hnRNPH3 and Stat3. The biogenesis and secretion of Cxcl14 and Cxcl12 are dependent on their receptor, Cxcr4, and on Ranbp2. Cxcl14 and Cxcl12 are inhibitory and stimulatory ligands of Cxcr4, the activation of which promotes Stat3 phosphorylation (P-Stat3) and nuclear import of P-Stat3 by importin β. Metalloproteinase 28 (Mmp28) is secreted from motoneurons and controls the homeostasis of chemokine signaling. Loss of Ranbp2 in cell bodies causes the uncoupling of crucial steps by the nuclear import and export receptors, and substrates thereof. This uncoupling leads to the nuclear retention of importin β and HDAC4, redistribution of exportin 1 between the nuclear and cytosolic compartments, and formation of cytoplasmic inclusions of Cxcl14-Cxcl12-Cxcr4 complexes, and latent and activated Stat3 (upper right). In motoneuronal axons of wild-type mice (lower left), there is free fatty acid oxidation by the mitochondria and liberation of fatty acids from PthCho. hnRNPH3 is localized to Schwann cells but is absent from the axoplasm. Cxcl14 likely exerts paracrine neuroglial signaling activities in concert with activated Stat3 and Mmp28. Loss of Ranbp2 promotes strong declines in PthCho and FFA. These declines lower ATP production from decreased FFA oxidation (bottom right). There is also an accumulation of *Cxcl14* mRNA, suppression of Cxcl14 synthesis and inhibitory signaling, and a decrease of Mmp28 levels caused by potential deficits in axoplasmic trafficking that lead to the paracrine upregulation of hnRNPH3 and activation of Stat3 in Schwann glia. Collectively, the dysregulations of these Ranbp2-dependent molecular processes promote the increase of the thickness of the axonal myelin sheath (g-ratio), the decrease of NCV and hypoactivity followed by hindlimb paralysis, respiratory distress and death of mice. PthCho, phosphatidylcholine; FFA, free fatty acid; hnRNP, hnRNPH3.

Emerging data support that MND, such as ALS, disrupts ribostasis, proteostasis and nucleocytoplasmic trafficking, and that modulators and effectors of the Ran GTPase cycle, which controls nucleocytoplasmic trafficking, act as disease modifiers of cytotoxicity of ALS-causing substrates and contribute to the phenotypic heterogeneity of MND (Jovičić et al., 2015; Freibaum et al., 2015; Zhang et al., 2015; Kim et al., 2013; Dickmanns et al., 2015; Cautain et al., 2015; Ramaswami et al., 2013; Ling et al., 2013; Xiao et al., 2015a,b; Boeynaems et al., 2016; Kinoshita et al., 2009; Zhang et al., 2006). However, the cause-effect mechanisms of components of the Ran GTPase cycle in motoneuron pathobiology were heretofore lacking. Ranbp2 plays a central role in the homeostasis of the Ran GTPase cycle (Patil et al., 2014; Cho et al., 2010; Villa Braslavsky et al., 2000; Vetter et al., 1999; Hamada et al., 2011; Ritterhoff et al., 2016) and controls the proteostasis of ALS-causing substrates, such hnRNPA2B1 (Cho et al., 2015b, 2014). In this study, we show that selective loss of *Ranbp2* in motoneurons in mice phenocopies prominent pathophysiological ALS traits, such as hindlimb paralysis, weight loss and respiratory distress, which culminate with the premature death of *SLICK-H::Ranbp2^flox/flox^* mice. Akin to other loss-of-function and gain-of-function genetic mouse models of ALS that affect components of the Ran GTPase cycle (Koppers et al., 2015; Peters et al., 2015; O'Rourke et al., 2015, 2016), loss of *Ranbp2* in motoneurons did not promote their degeneration. Although the reason(s) for the lack of this pathological outcome is not yet understood, it is possible that additional genetic or epigenetic modifying factors, such as aging, play a role in the degeneration of conspicuously large size motoneurons, as the death of mice 10 days after loss of *Ranbp2* in motoneurons will preclude the capture of a neurodegenerative trait. Regardless, this study reveals that the loss of Ranbp2 in motoneurons and appearance of pathophysiological ALS-like syndromes result from five different and possibly complementary mechanisms (see model in Fig. 10). These include: (1) the disruption of nucleocytoplasmic partitioning of Ran GTPase-dependent nucleocytoplasmic shuttling of nuclear transport receptors (e.g. exportin 1 and importin β) and substrates (e.g. HDAC4); (2) impairments in the chemokine signaling axis by Cxcl14/Cxcl12, Cxcr4, and latent and activated Stat3; (3) the post-transcriptional dysregulation of hnRNPH3 in paracrine and autocrine fashions in sciatic nerves and cell bodies, respectively; (4) the post-transcriptional dysregulation of Mmp28; and (5) the increase in FA-driven energy metabolism (decrease in RER) and the sharp decrease in free FA and PtdCho in sciatic nerves that likely contribute to declines of g-ratio and NCV in theses nerves.

The nucleocytoplasmic partitioning of Ran GTPase and its accessory partners, the nuclear export and import receptors exportin 1 and importin β, respectively, were profoundly disrupted in motoneurons in our mice. These effects were accompanied by the prominent dysregulation of nucleocytoplasmic partitioning and proteostasis of HDAC4, an accessory substrate of Ranbp2 (Kirsh et al., 2002; Cho et al., 2014; Scognamiglio et al., 2008). The loss of localization of these Ran GTPase cycle partners of Ranbp2 at the nuclear rim following loss of Ranbp2, support the contention that docking of exportin 1 and importin β to the ZnF and RBDs of Ranbp2, respectively (Singh et al., 1999; Villa Braslavsky et al., 2000; Vetter et al., 1999; Delphin et al., 1997), are crucial rate-limiting steps both in coupling nuclear and cytoplasmic transport for shuttling substrates, such as HDAC4, and in selectively controlling their proteostasis in motoneurons. These results extend support to our previous studies and the recent studies of others that found nucleocytoplasmic transport and proteostasis of substrates to be tightly coupled molecular processes, and that dysregulation of these mechanisms are co-opted by distinct neurodegenerative diseases in which pathological delocalization or accumulation of disease-prone substrates are pathological hallmarks of the illness (Cho et al., 2010; Woerner et al., 2016; Takahashi-Fujigasaki et al., 2006; Jovičić et al., 2015; Freibaum et al., 2015; Zhang et al., 2015; Li et al., 2012; Packham et al., 2015; Xiao et al., 2015a; Kinoshita et al., 2009; Zhang et al., 2006). Furthermore, this work lends support to the notion that Ranbp2 is physiologically required to exclude HDAC4 from the nuclear compartment. Loss of Ranbp2 not only promotes HDAC4 localization in the nuclear compartment, but also downregulates its proteostasis in motoneurons likely owing to dysregulation of HDAC4 proteolysis and sumoylation by the 26S proteasome and ubc9 activities, respectively, that are controlled by Ranbp2 (Kirsh et al., 2002; Cho et al., 2014; Scognamiglio et al., 2008; Yi et al., 2007).

Another unexpected finding of this study was that Ranbp2 controls the biogenesis of components of the chemokine signaling axis comprising Cxcl14/Cxcl12, Cxcr4 and Stat3. In particular, the strong transcriptional upregulation and translational downregulation of Cxcl14 by loss of Ranbp2 suggests that Ranbp2 uncouples the translation of *Cxcl14* mRNA in a fashion similar to that proposed for transcripts encoding some secretory proteins harboring alternative mRNA nuclear export (ALREX) elements within conserved sequences of the signal sequence-coding region (SSCR) (Mahadevan et al., 2013; Palazzo et al., 2007). In this regard, Ranbp2 was found to potentiate the synthesis of secreted proteins by the interaction of its zinc-finger-rich domain (ZnF) with ALREX elements (Mahadevan et al., 2013) and to control the nuclear export of eukaryotic translation initiation factor 4E (elF4E)-containing ribonuclear particles and elF4E-dependent mRNAs (Culjkovic-Kraljacic et al., 2012). Our studies indicate that the synthesis of Cxcl14 is required for the biogenesis of its serpentine receptor, Cxcr4, and its other ligand, Cxcl12, in the secretory pathway, as loss of Ranbp2 causes the prominent formation of intracellular Cxcr4^+^Cxcl12^+^ and Cxcr4^+^Cxcl14^+^ inclusions that are reminiscent to those of stress granules. The widespread intracellular colocalization of Cxcl12 and Cxcl14 with their receptor, Cxcr4, upon loss of Ranbp2, suggest that the biogenesis and secretion of Cxcr4, Cxcl12 and Cxcl14, and the localization of its effector, Stat3, are tightly coordinated and interdependent. This finding is analogous to mechanisms underlying M-opsin biogenesis, the only other known serpentine receptor whose biogenesis is chaperoned by Ranbp2 (Cho et al., 2014, 2015b; Ferreira et al., 1996, 1997) and 11-*cis* retinal (Zhang et al., 2008), which is the light-sensitive and chromophore ligand of opsins. Hence, the biogenesis of M-opsin and Cxcr4 likely co-opt Ranbp2-dependent mechanisms. Furthermore, it will be important to determine in future studies whether the role of Ranbp2 in the biogenesis of secreted proteins extends also to the secreted frizzle-related protein 3 precursor (Sfrp3/*Frzb*), the transcriptional levels of which were strongly suppressed in *SLICK-H::Ranbp2^flox/flox^* mice. Collectively, these findings provide novel insights into parallel mechanisms underlying the biogenesis of M-opsin and Cxcr4, the antagonizing secreted ligands Cxcl12 and Cxcl14, and, possibly, Sfrp3/*Frzb* and Wnt.

Additionally, findings from our study reveal that Thy1 motoneurons are a physiological source of Cxcl12/14 and Cxcr4. Notably, Cxcl12 and Cxcl14 are thought to act in opposition on GABAergic transmission by interneurons in the dentate gyrus but the underlying mechanisms are not understood (Banisadr et al., 2011). Hence, it is possible that suppression of Cxcl12 and Cxcl14 secretion by loss of Ranbp2 also depresses modulatory signaling to spinal and inhibitory GABAergic interneurons that shape motoneuron responses to excitatory inputs and motor performance (Gosgnach et al., 2006). Finally, the significance of the role of Ranbp2 in Cxcl12/Cxcl14•Cxcr4 signaling and biogenesis extends beyond the modulation of neuromotor microcircuitries. For example, Cxcr4 acts also as a main co-receptor for cellular entry of T-tropic strains of HIV-1 at late stages of infection and in individuals with acquired immune deficiency syndrome-associated dementia complex (ADC), who develop a spectrum of motor impairments and dementia (Bachis et al., 2006; Bleul et al., 1996). Notably, various activities of Ranbp2 are also implicated in the modulation of HIV-1 infection (Zhang et al., 2010; Schaller et al., 2011; Rasaiyaah et al., 2013). Hence, insights into how Ranbp2 controls the biogenesis of the Cxcl12/Cxcl14•Cxcr4 signaling complex will provide novel therapeutic opportunities to a spectrum of disease conditions.

Our prior studies identified the ALS-causing protein, hnRNPA2B1, as a substrate of Ranbp2, and hnRNPA2B1 proteostasis is dependent on the *cis-trans* prolyl isomerase activity of Ranbp2 (Cho et al., 2015b, 2014). In the present study, however, we found that loss of Ranbp2 in motoneurons causes dysregulation of hnRNPH3 proteostasis in a paracrine and autocrine fashion in Schwann cells surrounding the sciatic nerve and in cell bodies of motoneurons, respectively. The predilection of Ranbp2 for hnRNPH3 may reflect the lower abundance of hnRNPH3 compared with hnRNPA2B1 in the spinal cord. Hence, hnRNPH3 would be rendered more susceptible to downregulation by loss of proteostatic activities of Ranbp2 between the short time-frame of loss of Ranbp2 at d0 and the death of *SLICK-H::Ranbp2^flox/flox^* mice at d10.5. Alternatively, it is possible that hnRNPA2B1 and hnRNPH3 are controlled by cell type-dependent Ranbp2 activities. Regardless, although different members of hnRNP family of proteins participate in the splicing, nuclear export or intracellular trafficking of mRNAs or a combination of these functions (Mili et al., 2001; Carson and Barbarese, 2005), the biological functions specifically of hnRNPH3 (also called hnRNP 2H9) are largely obscure. Emerging data also indicate that a network of hnRNPs may be involved in ALS pathogenesis (Mohagheghi et al., 2016; Kim et al., 2013; Conlon et al., 2016). This study shows that hnRNPH3 is under multifaceted regulation by Ranbp2. For example, we found hnRNPH3 was downregulated in the spinal cord of *SLICK-H::Ranbp2 ^flox/flox^* mice at d0, whereas its levels were indistinguishable between genotypes at d10 by immunoblot analyses – even though the soma of motoneurons lacked conspicuous hnRNPH3 antigenicity. These results suggest that upon loss of Ranbp2, hnRNPH3 undergoes conformational changes that mask its antigenicity *in situ*. As hnRNPH3 was excluded from YFP^+^ motoneuronal axons but was found in surrounding Schwann cells and because hnRNPH3 proteostasis was deregulated in sciatic nerve, our findings suggest also the existence of a neuroglial signaling loop between axons of motoneurons and Schwann cells that controls hnRNPH3 proteostasis (Fig. 10). Notably, Cxcl12•Cxcr4 signaling and cyclophilin A, which has the highest homology to CY domain of Ranbp2, were linked to the stimulation of nuclear export of hnRNPA2 (Pan et al., 2008). Hence, it will be important to ascertain in future studies whether hnRNPH3 in Schwann cells is under paracrine control through competing stimulatory Cxcl12 and inhibitory Cxcl14 ligands of Cxcr4 that are secreted from motoneurons, and that ultimately lead to Stat3 activation in the sciatic nerve, as observed in this study.

Our previous investigations found that Ranbp2 selectively controls the expression and activation of a specific metalloproteinase, Mmp11, in cone photoreceptor neurons and RPE (Patil et al., 2014; Cho et al., 2013). In motoneurons, we found instead that loss of Ranbp2 promoted the post-transcriptional downregulation of Mmp28, a poorly understood Mmp isoform whose upregulation is linked to demyelination lesions, axonal-glial signaling and proinflammatory responses (Werner et al., 2008; Gharib et al., 2014). In this regard, our results suggest that a decline in Mmp28 expression reduces proinflammatory responses, as reflected by the lack of macroglial and microglial inflammatory markers in the spinal cord of *SLICK-H::Ranbp2^flox/flox^* mice, despite a robust transcriptional enhancement (fivefold) in the acute-phase responsive pro-inflamatory marker *Saa1* (Meek and Benditt, 1986). As with some metalloproteinases (Van Lint and Libert, 2007), it is also possible that downregulation of Mmp28 decreases the release or activation of signaling molecules, such as Cxcl12 and Cxcl14, to or from the extracellular matrix reservoir. Our mouse models of Ranbp2 will provide a foundation for dissecting these and other Mmp28 functions in axonal-glial signaling.

Finally, it should be emphasized that Ranbp2 pleotropic functions are apparently not required by all Thy1 neurons, as *SLICK-V* mice did not display overt phenotypes despite the loss of Ranbp2 expression across YFP^+^ neurons of the CNS in these mice. These cell type-dependent roles of Ranbp2 extend also to the biological functions of its domains, as motoneurons do not depend on biological activities of Ranbp2 that are required for the viability of other neural and supporting cell types, such as cone photoreceptor neurons and RPE (Cho et al., 2015b; Patil et al., 2014; Cho et al., 2014). For example, this study and other genetic complementation studies have shown that loss of activities in motoneurons of the domains of Ranbp2, such as CY, CLD, KBD and RBD2/3, do not lead to overt motor behavioral phenotypes (Cho et al., 2015b, 2014; Patil et al., 2014). Hence, other yet unknown activities of Ranbp2 are essential to motoneuronal functions. A strong candidate for this function is the zinc finger-rich domain (ZnF) of Ranbp2, the interaction of which with exportin 1 is likely crucial to control the coupling of the nuclear export of mRNAs complexed to hnRNPs for their dissasembly, translation or intracellular targeting in motoneurons (Singh et al., 1999; Mahadevan et al., 2013; Culjkovic-Kraljacic et al., 2012). Our Ranbp2 mouse models will permit future studies to identify specific biological and physiological activities of Ranbp2 that are crucial to motoneuron functions and they will provide insights into multifaceted pathobiological manifestations of MND controlled by Ranbp2.

## MATERIALS AND METHODS

### Mice

*Thy1-cre/ER^T2^-EYFP* (*SLICK-H*) mice were kindly provided by Guoping Feng (Massachusetts Institute of Technology, Cambridge, MA, USA; previously at Duke University) and *SLICK-V* mice were purchased from The Jackson Laboratory (Young et al., 2008). *SLICK-H* mice were crossed with *Ranbp2^flox/flox^* or *Ranbp2^+/+^* mice to produce *SLICK-H::Ranbp2^+/flox^* and *SLICK-H::Ranbp2^+/+^*, respectively. Then, *SLICK-H::Ranbp2^+/flox^* mice were crossed to *Ranbp^+/flox^* animals to generate *SLICK-H::Ranbp2^flox/flox^* on a mixed genetic background*.* The generation of transgenic *Ranbp2* mice, Tg^RBD2/3*-HA^, has been previously described (Patil et al., 2014). Hemizygous transgenic mice, Tg^RBD2/3*-HA^::*SLICK-H::Ranbp2^flox/flox^*, were generated by crossing *SLICK-H::Ranbp2^flox/flox^* with Tg^RBD2/3*-HA^::Ranbp2^flox/flox^. Tamoxifen (T5648; Sigma-Aldrich) was dissolved in corn oil with 2% of alcohol at 20 mg/ml and administered by oral gavage for 5 consecutive days (0.25 mg/g of body weight) to 4- to 6-week-old mice. Mice were reared in a pathogen-free transgenic barrier facility with a 12:12 h light:dark cycle (<70 lux; 6:00 AM-6:00 PM) under humidity- and temperature-controlled conditions, and given *ad libitum* access to water and chow diet 5LJ5 (Purina). Mice of either sex were examined by this study. All experiments were conducted with approved protocols from the Duke University Institutional Animal Care and Use Committee in accordance with NIH guidelines for the care and use of laboratory animals.

### Behavioral assays

Male and female *SLICK-H::Ranbp2^flox/flox^* and *SLICK-H::Ranbp2^+/+^* mice at 4-6 weeks of age were examined. Mice were housed in groups of three to five in a humidity- and temperature-controlled room with a 14:10 h light:dark cycle (lights on at 06:00 h) and provided with food and water *ad libitum*. Body weights were monitored at 0, 3, 6, and 10 days after tamoxifen or vehicle administration.

#### Open-field motor activity

Spontaneous activity in the open field was conducted over 15 min in an automated Omnitech Digiscan apparatus (AccuScan Instruments) (Taylor et al., 2008). Accuscan software was used to score the total distance traveled and vertical activity (beam breaks).

#### Rotorod performance

Balance and coordination were examined using an accelerating (4-40 rpm over 5 min) rotorod (Med-Associates) as described previously (Taylor et al., 2008) on days 0, 4, 8, 9 and 10 after tamoxifen or vehicle administration. Mice were given four successive 5 min trials that were separated by 30 min each. Trials were terminated when the mouse fell from the rod or at 300 s and were recorded as latency to fall.

#### Comprehensive laboratory animal monitoring system (CLAMS)

Mice were placed into the CLAMS apparatus (Columbus Instruments) after tamoxifen or vehicle administration and they were examined over 12 h (dark cycle) for locomotor activity, calorimetry, and feeding and drinking behaviors as described previously (Cawley et al., 2004). Indices of food (g) and water (ml) intake, volume of O_2_ intake (ml/kg/h), volume of CO_2_ output (ml/kg/h), and motor activity (total beam-breaks) were tabulated with Oxymax software (Columbus Instruments). This software calculated the respiratory exchange ratio (RER) for each mouse as a ratio of the volume of CO_2_ produced (ml/kg/h) to the volume of O_2_ consumed (ml/kg/h). Heat (kcal/kg/h) was derived from the equation for caloric value (CV)=3.815+1.232×RER, multiplied by the volume of oxygen consumed (VO_2_) adjusted to the body weight of the mice.

### Nerve conduction velocity

Nerve conduction velocity (NCV) was determined by electrically stimulating the sciatic nerve independently at two points a known distance apart, and dividing that distance by the difference in latency of the evoked electromyographic (EMG) response of the soleus muscle (Hirst et al., 1978). Under ketamine-xylazine anesthesia (100 mg/kg and 20 mg/kg), custom polyurethane cuff electrodes with two stainless steel contacts were placed on the nerve and supra-motor threshold biphasic stimulation delivered at 0.25 Hz. EMG was recorded at 100 kHz using insulated fine wire platinum-iridium electrodes, amplified 100× (ETH-255, CB Sciences) and band-pass filtered from 100-3000 Hz; a cross-correlation was used to calculate time lag (MATLAB, MathWorks). Temperature was measured via rectal thermometer (Physitemp Thermalert TH-8J) at 1 Hz and used to correct NCV by linear regression.

### Transmission electron microscopy

Mice were perfused intravascularly with 2.5% glutaraldehyde and 4% paraformaldehyde in 0.1 M sodium cacodylate buffer (pH 7.4). Sciatic nerves were dissected and fixed for 2 h at room temperature followed by 18 h at 4°C in the same fixative, postfixed in OsO_4_, and embedded in Araldite. Ultrathin sections were stained with uranyl acetate and lead citrate, and examined on a Phillips BioTwin CM120 electron microscope equipped with Gatan Orius and Olympus Morada digital cameras.

### Immunohistochemistry

After being anesthetized with ketamine/xylazine (100 mg/kg and 10 mg/kg of body weight, respectively), mice underwent cardiac perfusion with 2% paraformaldehyde in 1× PBS. Next, a dorsal laminectomy was performed in mice by removing the paravertebral muscles, transecting the vertebral arches and the ventral roots of the lumbar vertebrae, and dissecting the cord from the lumbar spinal column. Sciatic nerve and lumbar (L3-L6) spinal cord were incubated in 2% paraformaldehyde/1× PBS under gentle agitation for 4 h at room temperature, followed by 5% sucrose for 1 h and 30% sucrose until they sunk to the bottom of the dish at room temperature. Nerves were embedded and frozen in OCT compound and stored at −80°C. Nerve cryosections were collected on lysine-coated glass slides with a cryotome (Microm HM550), washed with 1× PBS, permeabilized and blocked in 0.2% Triton X-100/5% normal goat serum for 24 h before incubation with primary antibodies for 36-48 h followed by washing in 1× PBS. Sagittal brain cryosections of different regions were prepared as described previously (Cho et al., 2012). Anti-goat, anti-rabbit or anti-mouse AlexaFluor-488, AlexaFluor-594 or Cy5-conjugated secondary antibodies were incubated for 2 h. Hoechst (Invitrogen) was used to counterstain nuclei. Specimens were mounted on glass slides with Fluoromount-G (Southern Biotech) and images were acquired with a Nikon C1^+^ laser-scanning confocal microscope coupled with a LU4A4 launching base of four solid state diode lasers (407 nm/100 mW, 488 nm/50 mW, 561 nm/50 mW and 640 nm/40 mW) and controlled by Nikon EZC1.3.10 software (version 6.4).

### Morphometric analyses

Quantitation of the numbers and areas of the perikarya of YFP^+^ motoneurons of anterior horn cells of *SLICK-H::Ranbp2^+/+^* and *SLICK-H::Ranbp2^flox/flox^* mice was performed on confocal images taken from coronal sections of lumbar spinal cord 3-5. A minimum of four random cross-sections of the anterior horn per animal were analyzed (612 µm^2^ for counting motoneurons and 127 µm^2^ and 25 µm thick to measure perikarya areas, four animals per genotype). Image processing, measuring anterior horn regions and manual counting was carried out using NIKON Elements software version AR. The axon diameter and g-ratio were measured and calculated from electron microscopic images of cross-sections of sciatic and phrenic nerves. The g-ratio was calculated as the ratio between the area of the axon and the area of the axon with the myelin sheath using Metamorph 7.0 software (Molecular Devices). A minimum of 100 randomly chosen axons per image field from at least three non-overlapping images per mouse were used.

### Biochemical assays

Tissues were collected immediately after the mice were euthanized, snap frozen, placed on dry ice and stored at −80°C in a freezer. NP-40 extracts were prepared with Bullet blender (Next Advance, BBX24). Free fatty acids were measured as previously described (Cho et al., 2009) and as per manufacturer's instructions (Biovision). Activity of acetylcholinesterase was determined by the acetylcholinesterase activity colorimetric assay kit (Biovision) as directed by the manufacturer. The phosphatidylcholine colorimetric/fluorometric assay kit (Biovision) was used to measure the levels of phosphatidylcholine. Free and protein-conjugated ubiquitin levels were determined by the UbiQuant ELISA kit, as directed by the manufacturer (LifeSensors). Results were normalized against protein amounts in NP40-solubilized tissue extracts used for each assay. Protein concentrations of NP40-solubilized extracts were determined by the Bradford assay (BioRad).

### Antibodies

The following and previously characterized antibodies were used for immunofluorescence (IF) or immunoblots (IB): rabbit anti-Ranbp2 [8 μg/ml (IF), Ab-W1W2#10] (Cho et al., 2014), rabbit anti-hsc70 [1:3000 (IB), ENZO Life Science, ADI-SPA-816], mouse mAb414 against nuclear pore complex proteins Nup62, Nup153 and Nup358 [10 μg/ml (IF), Covance, MMS-120P], mouse anti-COMPV [1:100 (IF), Mitoscience, MS-502]; rabbit anti-Ran-GTP [1:100 (IF), a gift from Dr Ian Macara (Vanderbilt University); rabbit antiserum AR-12] (Richards et al., 1995), rabbit anti-CRM1 [1:50, (IF), Santa Cruz Biotechnology, SC-5595]; mouse Ran GTPase [1:100 (IF), 1:4000 (IB), BD Biosciences, 610341], mouse anti-importin β [mAb3E9, 1:100 (IF), a gift from Dr Steve Adams, Northwestern University, Evanston, IL, USA] (Chi et al., 1995), rabbit anti-HDAC4 [1:500 (IB), Santa Cruz Biotechnology, SC-11418], rabbit anti-Cxcl14 [1:100, (IF), 1:1000 (IB), Proteintech, 10468-1-AP], rabbit anti-Cxcl12 [1:100, (IF), 1:1000 (IB), Proteintech, 17402-1-AP], rat anti-Cxcr4 [1:100, (IF), R&D Systems, MAB21651], rabbit anti-STAT3 [1:100 (IF), 1:1000 (IB), Cell Signaling, 4904], rabbit phospho-STAT3 [1:100 (IF), 1:1000 (IB), Cell Signaling, 9145S], rabbit anti-GFAP [1:200, (IF), DAKO, Z0334], mouse anti-rat CD11b [1:100 (IF), AbD Serotec, mca275g], rabbit anti-hnRNPH3 [1:100, (IF), 1:1000 (IB), Proteintech, ARP40721], rabbit anti-Mmp28 [1:100, (IF), 1:1000 (IB), Proteintech, 18237-1-AP], anti-TDP-43 antibody [10 µg/ml, (IF), Proteintech, 10782-2-AP]. Alexa Fluor-conjugated secondary antibodies and Hoechst 33 342 were from Invitrogen.

### Immunoblotting

All tissues were snap frozen, placed on dry ice upon collection and stored at −80°C. Tissue homogenates were prepared as described previously with minor modifications (Cho et al., 2015a,b). Briefly, spinal cords were homogenized in radioimmune precipitation assay (RIPA) buffer with zirconium oxide beads (Next Advance, ZROB05) and a Bullet blender (Next Advance, BBX24) at 8000 rpm for 3 min, whereas nerves were homogenized with stainless beads (Next Advance, bead mixtures of SSB02 and SSB14B) at 9000 rpm for 2 min with a Bullet blender (Next Advance, BBX24). Protein concentrations of tissue homogenates were measured by the BCA method using BSA as the standard (Pierce). Equal amounts of homogenates (40 µg of spinal cord homogenates or 100 µg of sciatic nerve homogenates) were loaded and resolved in 7.5% SDS-PAGE Hoefer or 4-15% gradient Criterion gels (BioRad). Western blotting and antibody incubations were performed as described previously (Cho et al., 2015b, 2014). Blots were also reprobed for hsc70, the protein levels of which were unchanged between genotypes, for normalization and quantification. Unsaturated band intensities were quantified by densitometry with Metamorph v7.0 (Molecular Devices), and integrated density values (idv) of representative bands were normalized to the background and idv of hsc70 as described previously (Cho et al., 2015b, 2014).

### Two-dimensional difference in-gel electrophoresis (2D-DIGE) protein expression profiling

Sciatic nerves of mice after 10 days of vehicle (corn oil) or tamoxifen administration were solubilized in RIPA buffer followed by buffer exchange in two-dimensional lysis buffer [7 M urea, 2 M thiourea, 4% CHAPS, 30 mm Tris-HCl, (pH 8.8)] as described elsewhere (Cho et al., 2014). Homogenates were CyDye labeled, and global protein profiling between genotypes was conducted first by analytical and then preparative 2D-DIGE with Applied Biomics. Changes in protein expression levels with a twofold cut-off between genotypes were identified with DeCyder ‘in-gel’ analysis software. Protein spots of interest were picked for protein identification by tandem mass spectrometry (MALDI-TOF/TOF) and database search for protein ID. Data analyses and validation of mass spectrometry data by immunoassays were performed by the Ferreira laboratory.

### Total RNA isolation and qRT-PCR

Total RNA was isolated using TRIzol (Invitrogen) according to the manufacturer's instructions. RNA was reverse transcribed using SuperScript II First-Strand Synthesis System (Invitrogen). Quantitation of mRNA level with gene-specific primers was carried out with cDNA equivalent to 10 ng of total RNA, SYBR Green PCR Master Mix and ECO Real-Time PCR System (Illumina). The data were analyzed using Eco Real-Time PCR System Software version 4.0 (Illumina). The relative amount of transcripts was calculated by the ΔΔCT method and normalized to GAPDH.

### Next-generation sequencing using RNA-Seq

Sciatic nerves were incubated in RNA*later* (Ambion), snap frozen in liquid nitrogen and submitted to Otogenetics Corporation for RNA-Seq assays. Briefly, the integrity and purity of total RNA were assessed using Agilent Bioanalyzer or Tapestation and OD260/280. cDNA (1-2 μg) was generated from high-quality total RNA using the Clontech SMARTer cDNA kit (Clontech Laboratories, 634925), and adaptors were removed by digestion with *Rsa*I. The resulting cDNA was fragmented using Covaris or Bioruptor (Diagenode), profiled using Agilent Bioanalyzer or Tapestation, and subjected to Illumina library preparation using NEBNext reagents (New England Biolabs, E6040). The quality and quantity and the size distribution of the Illumina libraries were determined using an Agilent Bioanalyzer or Tapestation. The libraries were then submitted for Illumina HiSeq2000 or HiSeq2500 sequencing according to the standard operation. Paired-end 90 or 100 nucleotide (nt) reads were generated from RNAseq with a sequence depth of 45-70 million seq reads and checked for data quality using FASTQC (Babraham Institute, Cambridge, UK). The data were then subjected to analysis using the platform provided by DNAnexus or by the Center for Biotechnology and Computational Biology (University of Maryland, College Park, MD, USA) as previously described (Trapnell et al., 2012). Levels of individual transcripts were expressed as fragments per kilobase of exon per million fragments mapped (FPKM) and were obtained using Cufflinks. A *q*-value less than 0.05 was considered to be statistically significant.

### Statistics

The behavioral data were analyzed with SPSS 11 (SPSS) or GraphPad. The CLAMS data were analyzed by one-way ANOVA, while the body weight, open field and rotorod data were evaluated by two-way repeated measures ANOVA (RMANOVA). Bonferroni-corrected pair-wise comparisons were used as the post hoc tests. For NCV analysis, non-parametric Kruskal–Wallis test for group analysis was performed for temperature-corrected NCVs followed by a post hoc Wilcoxon signed-ranked test between each group (MATLAB R2015a). Differences in g-ratios and axonal diameters between groups were assessed with a *t*-test of difference between means using generalized estimating equations (GEE) to account for multiple nerves per mouse. The difference between groups adjusting for axonal diameter was assessed using generalized estimating equations with terms for group, axonal diameter and their interaction (SAS). The Mann–Whitney test rank-sum test was used to examine areas of perikarya in the motoneurons. For all other assays, Student's *t*-test for two groups was used. Data are reported as average values±s.d., except where otherwise specified. Differences among the groups were considered statistically significant when *P*≤0.05.
